# Pharmacologic management of HR+/HER2− mBC: a clinically oriented review

**DOI:** 10.3389/fonc.2025.1596634

**Published:** 2025-09-08

**Authors:** Elena Diana Chiru, Lina Sojak, Julia Landin, Marcus Vetter, Cvetka Grašič Kuhar

**Affiliations:** ^1^ University Center for Hematology and Oncology, Cantonal Hospital Baselland, Liestal, Switzerland; ^2^ Basel Medical University, Basel, Switzerland; ^3^ Department of Medical Oncology, Institute of Oncology Ljubljana, Ljubljana, Slovenia; ^4^ Faculty of Medicine, University of Ljubljana, Ljubljana, Slovenia

**Keywords:** hormone receptor positive breast cancer, endocrine resistance, metastatic breast cancer, CDK4/6 inhibitors, aromatase inhibitors

## Abstract

Breast cancer (BC) remains the most prevalent cancer among women worldwide, with hormone receptor-positive (HR+) and human epidermal growth factor receptor 2-negative (HER2-) subtypes representing approximately 75% of cases. Endocrine therapy (ET) has been foundational in HR+ BC treatment, significantly reducing recurrence and mortality rates, yet resistance to ET remains a critical challenge, particularly in the metastatic setting. Recent treatment advancements—especially CDK4/6 inhibitors (CDK4/6i) in first-line therapy, have reshaped management of HR+ HER2- mBC. Additionally, novel agents like selective estrogen receptor degraders (SERDs) and proteolysis-targeting chimeras (PROTACs), have proven to be effective against ER-resistance inducing mutations, such as ESR1, while poly ADP-ribose polymerase inhibitors (PARPi) showed targeted benefit in BRCA-mutated tumors. In breast cancer expressing AKT/PIK3CA pathway alterations, drugs like alpelisib, capivasertib, and inavolisib have recently been approved, demonstrating improved PFS in this specific patient population. Recent developments of antibody-drug conjugates (ADCs) have also extended therapeutic options to previously labeled HER2-negative tumors, with drugs like trastuzumab deruxtecan (T-DXd) demonstrating efficacy in newly emerged HER2-low and HER2-ultralow pathologic subgroups, extending median overall survival to almost 2 years. Most of these drugs have paved the way for personalized medicine and opened questions around optimal sequencing of ET and application of combination therapies, which continue to be investigated through clinical trials. This review seeks to highlight current and emerging treatment strategies addressing ET resistance to improve survival outcomes for HR+ mBC patients, emphasizing the need for personalized approaches.

## Introduction

With approximately 2.3 million new cases in 2022, breast cancer (BC) represents 25% of all cancer cases diagnosed in women worldwide. It accounts for nearly 12% of all cancer cases globally and 7% of cancer-related deaths annually (1). Despite recent treatment advances, most of these fatalities are due to development of therapy-resistant metastatic disease (2).

Most breast tumors are hormone-dependent, driven by estrogen dependent factors of growth and proliferation. Therefore, incidence of BC is approximately 150 times higher in women than in men, with a rapid increase in incidence observed in premenopausal women. Age-standardized incidence rates of premenopausal breast cancer are predominantly higher in high-income countries, while the incidence of postmenopausal BC has been rising significantly in countries undergoing economic and social transitions (3), possibly as a consequence of overlapping reduced prevention, increased prevalence of co-morbidities in older patients of transitioning countries and aging factors. Menopause experienced 10 years earlier than median age (52 years old) reduces the risk of BC by 35%. Similarly, other key moments in hormone development, such as age at menarche and timing of pregnancy play pivotal roles in BC occurrence. In a similar way, men who develop BC first experience gynecomastia, indicating an underlying endocrine disbalance, as 90% of male breast tumors are hormone receptor positive (HR+) (3, 4).

Assessment of HR status – estrogen receptor (ER) and progesterone receptor (PR), as well as human epidermal receptor 2 (HER2) status, and proliferation index (Ki67) is essential for diagnostic workup, molecular classification and risk stratification. The most commonly accepted subtypes have been determined based on immunohistochemical expression of HR and HER2, and have been classified into luminal A-like (HR+/HER2-, low Ki-67 expression), luminal B HER2-negative (HR+/HER2-, PR- or higher Ki67 expression), luminal B-like HER2+ (ER+/HER+, any level of Ki67, PR-/+), HER2 enriched (HR-, HER2+) and triple-negative or basal-like (HR-/HER2-) (5, 6).

In early stages of HR+/HER2- BC, endocrine therapy (ET) is the standard management, having proven efficacy in lowering mortality rate by one third (7). ET options have evolved from selective estrogen receptor modulator (SERM) tamoxifen to aromatase inhibitors (AIs). In a metanalysis conducted by the Early Breast Cancer Trialists Collaborative Group (EBCTCG) in 2011, 5 years of tamoxifen therapy in ER-positive BC patients lowered recurrence rates by half in the first 4 years (RR 0.53) and by one-third in years 5-9 (RR 0.68), with a sustained 30% reduction in BC mortality over 15 years (RR 0.71 in years 0-4, RR 0.66 in years 5-9, RR 0.68 in years 10-14) (8). Results from a EBCTCG metanalysis in postmenopausal patients, showed that 5 years of aromatase inhibitor (AI) treatment significantly reduced recurrence rates by about 30% when compared to tamoxifen (RR 0.70, 95% CI 0.64–0.77) and led to a 10-year BC mortality reduction of about 15% (RR 0.86, 95% CI 0.80–0.94). AIs are associated with fewer endometrial cancers, and are a better treatment alternative in patients with higher risk of thromboembolic events and those at risk of endometrial cancer. However, they pose a higher risk of bone fractures when compared to tamoxifen (9).

In premenopausal women on ovarian suppression and ET, the rate of BC recurrence was lower for women receiving AI than for women assigned to tamoxifen (RR 0.79, 95% CI 0.69–0.90, p=0.0005). The main benefit was seen in years 0–4 (RR 0.68), leading to a 3.2% reduction of recurrence at 5 years (10). However, in these younger patients no effect on mortality rate was observed.

### History

Benefits of ET in BC management have been acknowledged since the end of the 19^th^ century, when British surgeon George Beatson introduced the practice of oophorectomy to reduce soft tissue metastases in cases of advanced BC (11). However, it wasn’t until Clara Szego’s discovery of the ER pathway and its mediators, that ET became an established BC treatment option (12).

Although, antiestrogen strategies can now provide significant disease control, development of ET resistance constitutes one of the main challenges to date and is associated with late recurrence events (>5 years) (13).

Risk of recurrence is clinically assessed through features such as tumor size and lymph node status, and guides therapy decision-making. An earlier metanalysis conducted by the EBCTCG in HR+/HER2- patients showed in tumors harboring more than 3 positive lymph nodes, a distant recurrence risk at 5 years of 3%, in those with 1–3 nodes 2%, and in node negative tumors ≥ 20 mm 1%, while node-negative tumors smaller than 20 mm had a risk of 0.5-1% (13). Moreover, in an effort to guide de-escalation of CHT application, multigene prognostic assays such as Oncotype DX, MammaPrint, EndoPredict and Prosigna, have emerged in the past two decades to inform about risk of recurrence in early stage HR+ BC and have been incorporated into international oncologic guidelines to refine risk stratification, replacing clinical risk factors (14–17).

In the metastatic setting, however, first-line treatment outcomes do not appear to differ significantly between patients receiving chemotherapy (CHT) and those treated with ET alone (18). Moreover, patients with HR+ mBC are more likely to than those with early-stage BC to express an actionable mutation, specifically in the mitogen-activated protein kinases, such as MAPK/ERK (37% vs. 22%, respectively) and homologous recombinant deficiency (HRD) pathways (22% vs. 10%, respectively) (19).

Since 2012, a variety of ET-based combination treatments—typically using AIs or fulvestrant as the backbone—have become available. The first was the approval of the mTOR inhibitor everolimus, followed by the introduction of CDK4/6 inhibitors: palbociclib (2015), ribociclib (2018), and abemaciclib (2018). Subsequent approvals included PI3K inhibitors such as alpelisib (2019) (20), and more recently inavolisib (21), as well as AKT inhibitors, such as capivasertib (22). Additionally, PARP inhibitors—olaparib (2018) and talazoparib (2023)—have been approved for patients with germline *BRCA1* or *BRCA2* mutations (19) – see **Tables 1a**, **b**.

**Table 1a:** Therapeutic agents in metastatic breast cancer grouped by mechanism and target.

Group	Drug name	Mechanism of action	Target
Aromatase Inhibitors (AIs)	Letrozole, Anastrozole, Exemestane	Inhibit aromatase enzyme, reducing estrogen production	Aromatase enzyme
SERMs	Tamoxifen	Modulates ER activity, blocking signaling on BC cells	ER
SERDs	Fulvestrant, Elacestrant, Camizestrant, Giredestrant	Degrade ER, leading to reduced estrogen signaling	ER
CDK4/6 Inhibitors (CDK4/6i)	Palbociclib, Ribociclib, Abemaciclib	Inhibit cyclin-dependent kinases 4 and 6, preventing cell cycle progression	CDK4/6
PI3K/AKT/mTOR Pathway Inhibitors	Alpelisib, Capivasertib, Everolimus, Inavolisib	Target PI3K/AKT/mTOR signaling pathway, reducing tumor growth and survival	PI3K, AKT, mTOR
PARP Inhibitors	Olaparib, Talazoparib	Inhibit PARP enzymes, leading to accumulation of DNA damage in cancer cells with BRCA mutations	Poly(ADP-ribose) polymerase (PARP)
Antibody-Drug Conjugates (ADCs)	Trastuzumab deruxtecan (T-DXd), Sacituzumab govitecan, Datopotamab deruxtecan	Combine monoclonal antibodies with cytotoxic agents to target and kill cancer cells	HER2, TROP2
PROTACs (Proteolysis-Targeting Chimeras)	ARV-471	Induce degradation of ER via the ubiquitin-proteasome pathway	Estrogen receptor (ER)
ShERPAs (Selective Estrogen Receptor Partial Agonists)	Experimental drugs (e.g., Xiong et al.)	Partial agonists of ER with activity against tamoxifen-resistant breast cancer	Estrogen receptor (ER)
Immunotherapy	Pembrolizumab, Avelumab	Block PD-1/PD-L1 interactions, enabling immune system to recognize and attack cancer cells	PD-1/PD-L1

ER, Estrogen Receptor; BC, Breast Cancer; SERMs, Selective Estrogen Receptor Modulators; SERDs, Selective Estrogen Receptor Degraders; CDK4/6i, Cyclin-Dependent Kinase 4/6 Inhibitors; PI3K/AKT/mTOR, Phosphatidylinositol 3-Kinase/Protein Kinase B/Mammalian Target of Rapamycin Pathway; PARP, Poly (ADP-Ribose) Polymerase; ADC, Antibody-Drug Conjugates; PROTACs, Proteolysis Targeting Chimeras; ShERPAs, Selective Human Estrogen Receptor Partial Agonists; ER, Estrogen Receptor; HER2, Human Epidermal Growth Factor Receptor 2; PI3K, Phosphatidylinositol 3-Kinase; AKT, Protein Kinase B; mTOR, Mammalian Target of Rapamycin; CDK4/6, Cyclin-Dependent Kinase 4 and 6; PARP, Poly (ADP-Ribose) Polymerase; PD-L1, Programmed Death-Ligand 1; Trop-2, Trophoblast Cell Surface Antigen 2.

**Table 1b:** List of clinical trials and drugs presented in this paper, grouped by class.

Name of Study/Paper	Study Phase	Company	Years Conducted	Population	Drug Investigated	Control/Comparator	PFS	OS (Test vs. Control)	ORR	CBR	Safety Profile	FDA Approval Date	EMA Approval Date	Guideline Recommendations
Selective Estrogen Receptor Modulators (SERMs)														
NATO (1988)	3	AstraZeneca	1976-1980	Postmenopausal HR+ advanced/mBC	*Tamoxifen*	Placebo					Hot flashes, nausea, vaginal discharge, menstrual irregularities, endometrial changes	Dec-77	Prior to EMA formation, approved through national regulatory agencies	
ASTRRA (2018)	3	ICI Pharmaceuticals (now part of AstraZeneca)	2009-2014	Premenopausal women with ER+ BC	*Tamoxifen + Ovarian Function Suppression (OFS*)	Tamoxifen alone	5-year 89.4% vs. 85.1%, HR 0.71	96.5% vs. 95.3%, HR 0.78	N/A	N/A	50% hot flashes (5% grade 3), 40% arthralgia (3% grade 3), 35% fatigue, 30% nausea, 25% headache	N/A	N/A	Premenopausal pts. with ER+ HER2- BC, adjuvant and mBC
MORE (1999)	3	Eli Lilly	1994-1998	Postmenopausal women with osteoporosis	*Raloxifene*	Placebo	N/A	N/A	n/A	72% reduction in incidence of BC	25-28% hot flashes, 5.5% leg cramps	Dec-97	Aug-98	Osteoporosis, BC risk reduction in postmenopausal women
ELAINE 1 (2023)	2	Pfizer/Sermonix Pharmaceuticals	2019-2022	Postmenopausal pts. with HR+ HER2- mBC and ESR1 mut	*Lasofoxifene*	Fulvestrant	6.04 mo. vs. 4.04 mo., HR	N/A	13.2% vs. 2.9%	36.5% vs. 21.6%; DNA ESR1 mutant allele fraction, with an 82.9% drop from baseline at week 8 compared to 61.5% in the fulvestrant arm	27.5% nausea, 23.5% fatigue, 21.6% arthralgia, 21.6% hot flushes, 15.7% constipation, 15.7% hypertension, 15.7% cough	N/A	N/A	Since 2009 for prevention and -treatment of osteoporosis
Aromatase Inhibitors (AIs)														
Letrozole (1996)	3	Novartis	1993-1995	Advanced BC patients, who had progression after antiestrogen therapy	*Letrozole*	Megestrol acetate	6.1 mo. vs. 4.2 mo., HR 0.73	26 mo. vs. 22 mo., HR 0.82	24% vs. 16%	49% vs. 38%	Hot flashes, nausea, fatigue, arthralgia, headache	Jul-97	Jul-96	First line option for HR+ HER2- advanced BC
North American Multicenter Randomized Trial (1996)	3	AstraZeneca	1993-1995	Postmenopausal women with HR+ advanced BC	*Anastrozole*	Megestrol acetate	5.7 mo. vs. 3.7 mo., HR 0.79	26.7 mo. vs. 22.5 mo., HR 0.78	21% vs. 17%	59% vs. 46%	Hot flashes, nausea, fatigue, bone pain, headache, thromboembolic events, fractures	Dec-95	Jul-96	First line in HR+, HER2- advanced BC
Exemestane Study Group Trial (2000)	3	Pfizer	1995-1997	Postmenopausal with ER+ advanced BC after progression on tamoxifen	*Exemestane*	Megestrol acetate	4.7 mo. vs. 3.8 mo., HR 0.69	20.4 mo. vs. 17.4 mo., HR 0.83	15% vs. 12.4%	44.2% vs. 33.6%	Hot flashes, nausea, fatigue, increased sweating, arthralgia, peripheral edema (grade 3), dyspnea and chest pain (grade 3)	Oct-99	Mar-00	Postmenopausal women with HR+ mBC after progression on non-steroidal Ais or tamoxifen
Selective Estrogen Receptor Degraders (SERDs)														
FALCON (2016)	3	AstraZeneca	2012-2016	Postmenopausal women with HR+advanced BC	*Fulvestrant*	Anastrozole	16.6 mo. vs. 13.8 mo., HR 0.79	44.8 mo. vs. 42.7 mo., HR 0.97	46.1% vs. 44.9%	79.5% vs. 77.2%	16.7% arthralgia, 11.4% hot flushes, 10.5% nausea	Apr-02	Aug-04	First line advanced BC if no previous ET, second line after progression on ET
EFECT (2008)	3	Astra Zeneca	2008	HR+/HER2- mBC progressing or recurring after NSAI	Fulvestrant	Exemestane	3.7 mo. for both groups	Not significantly different between groups	7.4% vs. 6.7%	32.2% vs. 31.5%	Well tolerated	N/A	N/A	Both fulvestrant and exemestane are recommended options for postmenopausal women with HR+ mBC after progression on NSAI
FIRST	2	AstraZeneca	2004-2010	HR+/HER2- mBC, no prior ET for advanced disease	*Fulvestrant 500 mg*	Anastrozole	23.4 mo. for fulvestrant vs. 13.1 mo.	54.1 mo. (fulvestrant) Vs. 48.4 mo.	72.5% vs. 67%	36% vs. 35.5%		Aug-10	Mar-10	Recommended as a first-line treatment option for postmenopausal women with HR+ advanced BC
CONFIRM (2010)	3	AstraZeneca	2005-2007	Postmenopausal women with HR+ advanced BC	*Fulvestrant 500 mg*	Fulvestrant 250 mg	6.5 mo. vs. 5.5 mo., HR 0.80	26.4 mo. vs. 22.3 mo., HR 0.81	9.1% vs. 10.2%	45.6% vs. 39.6%	Injection-site pain 11.6%, nausea 9.7%, bone pain 9.4%	Sep-10	Mar-10	HR+ mBC in combination with CDK4/6i or monotherapy in first line, in second or subsequent lines after progression on ET, monotherapy or combination with CDK4/6i, mTORi, PI3Ki
EMERALD (2022)	3	Radius Health (Menarini Group)		2019-2021	*Elacestrant*	Standard ET	7.3 mo. vs. 3.1 mo.	HR in the overall population 0.75, in the ESR1 mut. 0.59	7.4% vs. 3.5%; in the ESR1 mut 8.2% vs. 3.1%	31.5% vs. 21.1%; in the ESR1 mut 32.5% vs. 14%	41% musculoskeletal pain (grade 3/4 7%), nausea 35% (2.9% grade 3/4), 26% vomiting, 15% inappetence	Jan-23	Sep-23	Second or subsequent line after progression on ET
SERENA-2 (2022)	2	AstraZeneca	2020-2022	HR+/HER2- advanced BC	*Camizestrant*	Fulvestrant	7.7 mo. vs. 3.7 mo.	N/A	75mg 15.7%, 150 mg 20.3% vs. fulvestrant 11.5%	75 mg 42.4%, 150 mg 33.3% vs. fulvestrant 28%	Photopsia 75 mg 12.2%, 150 mg 24.7%; bradycardia 75 mg 5.4%, 150 mg 26%	N/A	–	N/A
SERENA-4	3	AstraZeneca	2021-ongoing	HR+/HER2- who have not received systemic treatment for advanced disease	*Camizestrant + palbociclib*	Anastrozole + palbociclib	N/A	N/A	N/A	N/A	Expected class effects: diarrhea, fatigue, nausea, neutropenia, low discontinuation	N/A	N/A	N/A
SERENA-6	3	AstraZeneca	2021–ongoing (interim data Feb 2025)	Postmenopausal women with ER+/HER2− advanced BC with emergent ESR1 mutation on AI + CDK4/6i	*Camizestrant + same CDK4/6 inhibitor (palbo/ribo/abema)*	Continue AI + same CDK4/6i	16 mo. vs 9.2 (HR 0.44, p<0.00001)	immature	Not yet available	60% grade 3 or higher in camizestrant arm vs. 46% with AI: neutropenia (45% vs. 34%), anemia (5% vs. 5%) and leukopenia (10% vs. 3%)	Consistent with known CDK4/6i + SERD AEs; no new safety signals; well tolerated	N/A	N/A	Not yet included; mutation-guided treatment strategy under evaluation
acelERA (2024)	2	Genentech	2020-2022	ER+, HER2-, advanced BC	*Giredestrant*	Physicians’ Choice of ET	2.9 mo. vs. 2.8 mo., HR 0.81	16.3 mo. vs. 15.6 mo., HR 0.91	9.8% vs. 7.2%	29.5% vs. 25.5%	Diarrhea 8.7%	N/A	N/A	N/A
EMBER-3 (2024)	3	Eli Lilly	2021-2024	HR+/HER2- advanced BC	*ImIunestrant*	Investigator’s choice (fulvestrant/exemestane)	In pts with ESR1 mutation: 5.5 mo. Vs. 3.8 mo.	immature	Imlunestrand vs. investigator’s choice 15.2% vs 14.6%; Imlunestrant + Abemaciclib vs. Imlunestrant 34.8% vs. 15.2%	N/A	imlunestrant: fatigue (23%), diarrhea (21%), nausea (17%), arthralgia (14%), elevated AST (13%), back pain (11%), elevated ALT (10%), anemia (10%), constipation (10%)	no	no	no
							In all pts: 5.6 mo vs. 5.5 mo				imlunestrant + abemaciclib: diarrhea (86% any grade, 8% grade ≥3), nausea (49% any grade, 2% grade ≥3), neutropenia (48% any grade, 20% grade ≥3), anemia (44% any grade, 8% grade ≥3), fatigue (39% any grade, 5% grade ≥3), vomiting (31% any grade, 1% grade ≥3), leukopenia (26% any grade, 4% grade ≥3), hypercreatininemia (22% any grade, 1% grade ≥3)			
							Imlunestrant + Abemaciclib vs. Imlunestrant alone: 9.4 mo. vs. 5.5 mo.							
AMEERA-3 (2022)	2	Sanofi	2019-2022	HR+/HER2- mBC progressed on or after ET	*Amcenestrant*	Physicians’s Choice of ET	3.6 mo. for amcenestrant vs. 3.7 mo. for the control group, with a hazard ratio of 1.051	immature	N/A	N/A	Grade ½ nausea (14.0% vs 4.1%), vomiting (8.4% vs 1.4%), hot flush (8.4% vs 7.5%), asthenia (7.0% vs 1.4%), fatigue (5.6% vs 6.1%), and injection site pain (0% vs 6.8%). Grade ≥3 in 4.9% amcenestrant vs. 0.7% in the control	N/A	N/A	Did not meet primary endpoint
Cyclin Dependent Kinase 4/6 Inhibitors (CDK4/6i)														
PALOMA-3 (2016)	3	Pfizer	2013-2015	HR+/HER2- mBC after ET failure	*Fulvestrant + Palbociclib*	Fulvestrant + Placebo	9.5 mo. vs. 4.6 mo.	mo.S 34.8 mo. vs. 28 mo.; HR 0.81; 6 year OS 19% vs. 12.9%	24.6% vs. 10.9%	67% vs. 40%	Neutropenia (grade 3 55%), leukopenia (grade 3 28%), anemia (grade 3 5%), fatigue (38%), nausea (29%)	Feb-15	Nov-16	HR+/HER2- mBC
PACE-1 (2022)	2	Pfizer	2017–2022	HR+/HER2- mBC after progression on prior CDK4/6 inhibitor and ET	*Fulvestrant + Palbociclib ± Avelumab*	Fulvestrant alone	4.6 mo. (Fulvestrant + Palbociclib) vs. 4.8 mo. (Fulvestrant alone); mPFS: 8.1 mo.(Fulvestrant + Palbociclib + Avelumab)	24.6 mo. (Fulvestrant + Palbociclib) vs. 27.5 mo. (Fulvestrant alone); Median OS: 42.5 mo. (Fulvestrant + Palbociclib + Avelumab)			Neutropenia mit Palbociclib; immune-related adverse events, such as thyroid dysfunction and pneumonitis with avelumab	N/A	N/A	Continuation of palbociclib beyond progression is not recommended; further research is needed for the triplet combination with avelumab
(Palbociclib After CDK and Endocrine Therapy)														
PADA-1 (2021)	3	Pfizer	2017–2021	HR+/HER2- mBC	*Fulvestrant + Palbociclib*	AI + palbociclib	11.9 mo. vs. 5.7 mo. HR 0.61	immature	N/A	N/A	Grade ≥3 neutropenia: 44.3% (Fulvestrant + Palbociclib) vs. 41.7% (AI + Palbociclib); Grade ≥3 lymphopenia: 4.5% vs. 3.6%	N/A	N/A	Monitoring for ESR1 mutations in ctDNA to guide early switch to fulvestrant in combination with palbociclib upon detection of mutations
MONARCH 2 (2017)	3	Eli Lilly	2014-2017	HR+ mBC progressed on ET	*Fulvestrant + Abemaciclib*	Fulvestrant + Placebo	16.4 mo. vs. 9.3 mo.	46.7 mo. vs. 37.3 mo., HR 0.757	48.1% vs. 21.3%	72.2% vs. 51.5%	Diarrhea 86% (grade 3 13%), 46% neutropenia (grade 3 32%), 39% fatigue, 28% leukopenia, 26% nausea	Sep-17	Sep-18	Adjuvant with ET for N+ early, high risk BC; 1st line + ET in HER2- mBC; 2nd line in combination with fulvestrant for HER2- mBC, after progression on ET
MONARCH 3 (2019)	3	Eli Lilly	2014-2023	HR+/HER2- mBC	*Abemaciclib + nonsteroidal aromatase inhibitor (NSAI)*	nsAI + Placebo	28.2 mo. vs. 14.8 mo. HR 0.540	67.1 mo. vs. 54.5 mo. HR 0.754	61.0% vs. 45.5%	79.6% vs. 69.6%	Diarrhea (81.3%), neutropenia (46.0%), fatigue (39.9%), infections (36.6%), nausea (35.2%)	Sep-17	Sep-18	First-line treatment for HR+, HER2-negative advanced or mBC in postmenopausal women
monarchE (2020)	3	Eli Lilly	2017–2023	high-risk, node-positive, early-stage HR+, HER2-negative BC	*Abemaciclib + ET*	ET + placebo	4-year iDFS: 85.8% vs. 79.4%; HR 0.664	Immature data	N/A	N/A	Diarrhea, neutropenia, fatigue, leukopenia	Oct-21	Nov-21	Adjuvant treatment for high-risk, node-positive, early-stage HR+, HER2-negative BC
MONALEESA-2 (2016)	3	Novartis	2013-2016	HR+/HER2- mBC	*Letrozole + Ribociclib*	Letrozoele + Placebo	25.3 months vs. 16.0 months	63.9 mo. vs. 51.4 mo., HR 0.76	N/A	N/A	Neutropenia (75.8% any grade, 59.3% grade 3/4), leukopenia (33.4% any grade, 14.3% grade 3/4), nausea (51.5%), fatigue (36.5%), diarrhea (35.0%), alopecia (33.2%)	Mar-17	08/207	First-line treatment for HR+, HER2– advanced BC in postmenopausal women
ABIGAIL	2	Eli Lilly	2021-2024	HR+/HER2- mBC	*Abmaciclib + Letrozole/Fulvestrant*	Paclitaxel 12 weeks followed by Abemaciclib + ET	Not yet available	Not yet available	12-week ORR 58.8% in arm A and 40.2% in arm B (OR: 2.12 [95% CI, 1.13–3.96]; p=0.019).	N/A	Diarrhea (68% any grade, ca. 10–12% grade 3/4), asthenia/fatigue (37% any grade, ca. 5% grade 3/4), nausea (25% any grade), neutropenia (27% any grade, 9% grade 3/4), anemia (16% any grade), alopecia (5%)	Oct-21	Nov-21	Adjuvant treatment for high-risk, node-positive, early-stage HR+, HER2-negative BC
MONALEESA-3 (2018)	3	Novartis	2015-2017	HR+/HER2- mBC	*Fulvestrant + Ribociclib*	Fulvestrant + Placebo	20.5 mo. vs. 12.8 mo.	53.7 mo. vs. 41.5 mo., HR 0.73;	40.9% vs. 28.7%	69% vs. 60%	Grade 3 neutropenia 57.1%, hepatobiliary toxicity grade 3 or mo.re 13.7%, QT prolongation grade 3 3.1%, grade3 respiratory disorder 2.3%, ILD 0.2%	Mar-17	Aug-17	Adjuvant + AI in Stage II and III early BC, first line in HER2- mBC + ET; second line + fulvestrant in mBC after progression on ET
MONALEESA-7 (2019)	3	Novartis	2014–2019	ER+, HER2-, advanced BC	*Ribociclib + ET (goserelin + tamoxifen or NSAI)*	Placebo + ET	23.8 mo. vs. 13.0 mo.	58.7 mo. vs. 48.0 mo., HR 0.76	51% vs. 36%	80% vs. 67%	Neutropenia (76% any grade, 61% grade 3/4), leukopenia (31% any grade, 14% grade 3/4), hot flushes (34%), nausea (32%), arthralgia (30%)	Jul-18	Aug-18	First-line treatment for HR+, HER2– advanced BC in premenopausal women
NATALEE (2023)	3	Novartis	2019–2024	HR+, HER2-negative early-stage BC (Stage II and III)	*Ribociclib + ET*	Placebo +ET	3-year iDFS: 90.4% vs. 87.1%; HR 0.748	immature	N/A	N/A	Neutropenia (44.3%), liver-related adverse events (8.6%), QT prolongation (1.0%), fatigue, nausea	Sep-24	Nov-24	Adjuvant treatment for patients with HR+, HER2-negative early-stage BC, including those with node-negative disease
TRINITI-1 (2021)	01-Feb	Novartis	2016-2020	HR+, HER2- advanced BC after progression on a CDK4/6i	*Ribociclib + Everolimus + Exemestane*	None (Single-arm study)	5.7 mo.	immature	7.70%	at 24 weeks: 41.1%	neutropenia (69.2%) and stomatitis (40.4%)	N/A	N/A	Further investigation of CDK4/6i combined with PI3K/AKT/mTOR pathway targeting in HR+/HER2− advanced BC after CDK4/6i progression
pionERA (ongoing)	1/2	Genentech	Since 2024	ER+, HER2-, advanced BC resistant to adjuvant ET	*Giredestrant + CDK 4/6i*	Fulvestrant + CDK 4/6i	N/A	N/A	N/A	N/A	N/A	N/A	N/A	N/A
RIGHT Choice (2022)	2	Novartis	2019-2022	Premenopausal women with aggressive HR+/HER2- advanced BC	*Ribociclib + ET*	Physician’s choice combination CHT	24 mo. vs. 12.3 mo., HR 0.54	N/A	66.1% vs. 61.8%, HR 0.76	81.3% vs. 74.5%	Neutropenia 61.6% (60.7% grade 3/4), leukopenia 35.7% (14.3% grade 3/4), 26.8% nausea, 23.2% fatigue	Mar-17	Aug-17	In first line adjuvant treatment of pts. with HR+, HER2- high risk patients; in combination with fulvestrant for mBC either first line or after disease progression on ET
Young PEARL	2	Pfizer	2015-2019		*Palbociclib + Exemestane + GnRH Agonist*	Capecitabine	20.1 mo. vs. 14.4 mo.	N/A	37% vs. 34%		Higher incidence of neutropenia in palbociclib arm	N/A	N/A	Not included
				Premenopausal women with HR+ HER2- mBC previously treated with tamoxifen										

PEARL (2020)	3	GEICAM Spanish BC Geoup	2014-2018	Postmenopausal women with HR+/HER2- mBC after progression on AI	*Palbociclib and ET (exemestane and fulvestrant)*	Capecitabine	7.5 mo. vs. 10 mo., HR 1.09	N/A	26.7% vs. 33.3%	50% vs. 53.3%	57.4% neutropenia (palbociclib + exemestane), 55.7% (palbociclib + fulvestrant)	N/A	N/A	Second line/subsequent in HR+ HER2- mBC with progression/relapse after prior ET
SONIA (2024) (Selecting the Optimal positioN of CDK4/6i in Advanced BC)	3	Investigator-led (Dutch Breast Cancer Research Consortium)	2017–2024	HR+/HER2- mBC	*CDK 4/6i + AI*	ET alone followed by CDK4/6i at progression	No significant difference in PFS2 between groups	No significant difference in OS between groups	N/A	N/A	First-line CDK4/6 inhibitors increased toxicity by 74% compared to second-line us	N/A	N/A	CDK4/6i may be equally effective when given after first-line ET rather than upfront; delayed use reduces toxicity and costs
MAINTAIN (2023)	2	Investigator-Initiated	2016–2021	HR+/HER2- mBC who progressed on prior CDK4/6i and ET	Ribociclib + Switch ET (Fulvestrant or Exemestane)	Placebo + Switch ET	5.29 mo. vs. 2.76 mo. HR 0.57	immature	N/A	N/A	Higher incidence of neutropenia in the ribociclib arm; other adverse events were manageable	N/A	N/A	Suggests potential benefit of continuing CDK4/6i with ribociclib after progression on prior CDK4/6i
Mechanistic Target of Rapamycin Inhibitors (mTORi)														
BOLERO-2 (2012)	3	Novartis	2010-2011	Postmenopausal women with HR+ advanced BC	*Everolimus + Exemestane*	Placebo + Exemestane	7.8 mo. vs. 3.2 mo., HR 0.45	N/A	12.6% vs. 1.7%	50.5% vs. 25.5%	Stomatitis 56% (grade 3 8%), rash 36%, fatigue 33%, diarrhea 30%, inappetence 29%, grade 3 anemia 6%, grade 3 dyspnea 4%, grade 3 hyperglycemia 4%, grade 3 pneumonitis 3%	Mar-09	Oct-10	In subsequent lines of therapy in HR+, HER2-, mBC after prior ET
MANTA (2017)	2	AstraZeneca	2014-2016	HR+/HER2- mBC progressed after AI	*Fulvestrant + Everolimus or Vistusertib*	Fulvestrant + Everolimus vs Fulvestrant Alone	-Fulvestrant + Everolimus: 12.3 mo. - Fulvestrant + Daily Vistusertib: 7.6 mo- - Fulvestrant + Intermittent Vistusertib: 8.0 mo.- Fulvestrant Alone: 5.4 months	immature	Fulvestrant + Everolimus: 8%​	Fulvestrant + Everolimus: 50%​	stomatitis, rash, and fatigue	Mar-09	Jun-12	N/A
									Fulvestrant + Daily Vistusertib: 7%​	Fulvestrant + Daily Vistusertib: 37%​				
									Fulvestrant + Intermittent Vistusertib: 5%​	Fulvestrant + Intermittent Vistusertib: 33%​ Fulvestrant Alone: 32%				
									Fulvestrant Alone: 7%					
Poly(ADP-ribose) Polymerase Inhibitors (PARPi)														
OlympiAD (2017)	3	AstraZeneca	2014-2015	HER2-negative mBC with germline BRCA mutations	*Olaparib*	CHT	7 mo. vs. 4.2 mo., HR 0.58	19.3 mo. vs. 17.1 mo., HR 0.90	59.9% vs. 28.8%	71.5% vs. 42.9%	Nausea 58%, anemia 40% (16% grade 3/4), fatigue 37% (4% grade 3), vomiting 30%, diarrhea 21% (grade 3 1%)	Jan-18	Mar-19	In patients with HER2- mBC, with a BRCA germline mutation, previously treated with ET, or in the case of HR- previously treated with CHT
EMBRACA (2018)	3	Pfizer	2013-2015	HER2-negative advanced BC with germline BRCA mutations	*Talazoparib*	CHT	8.6 mo. vs. 5.6 mo., HR 0.54	19.3 mo. vs. 19.5 mo., HR 0.85	62.6% vs. 27.2%	68.6% vs. 36.1%	Anemia 53% (39% grade 3/4), fatigue 50%, nausea 48% (grade 3 1%), neutropniea 35% (21% grade 3/4), thrombocytopenia 27% (15% grade 3/4)	Oct-18	Jun-19	In patients with HER2- BRCA mutated locally advanced or mBC, previously treated with ET or, for those HR+, with CHT
Phosphatidylinositol 3-Kinase Inhibitors and Ak strain Transforming Inhibitors (PIK3CAi/AKTi)														
SOLAR-1 (2019)	3	Novartis	2015-2017	HR+/HER2- mBC with PIK3CA mutation	*Alpelisib + Fulvestrant*	Fulvestrant + Placebo	11.0 mo. vs. 5.7 mo.	39.3 mo. vs. 31.4 mo., HR 0.86	26.6% vs. 12.8%	61.5% vs. 45.3%	63.7% hyperglycemia (36.6% grade 3/4), skin rash 35.6% (9.9% grade 3), 57.7% diarrhea (6.7% grade 3)	May-19	Jul-20	Second line after progression on ET in PIK3CA-mutated HR+ mBC
CAPItello-291 (2023)	3	AstraZeneca	2020-2022	HR+/HER2- mBC, post-CDK4/6i	*Capivasertib + Fulvestrant*	Placebo + Fulvestrant	7.2 mo. vs. 3.6 mo.	39.3 mo. vs. 31.3 mo., HR 0.70	22.9% vs. 12.2%; in the biomarker altered pts 28.8% vs. 9.7%	51% vs. 39%; in the biomarker altered pts 56% vs. 40%	72% diarrhea (9.3% grade3/4), 33% nausea, 30% fatigue, 28% hyperglycemia (2.3& grade 3/4), grade 3 rash 12.1%	Nov-23	Jun-24	Second line in ET resistant, PIK3CA mut advanced BC
CAPItello-292 (2021)	1b/3	AstraZeneca	2021–2029​	HR+/HER2- mBC	*Capivasertib + CDK4/6 inhibitors (ribociclib, palbociclib, or abemaciclib) +fulvestrant*	CDK4/6 inhibitors + fulvestrant	ongoing	ongoing	N/A	N/A	N/A	Nov-23	Jun-24	Second line in ET resistant, PIK3CA mut advanced BC
FAKTION (2022)	2	AstraZeneca	2015-2018	PI3K/AKT pathway alterations	*Capivasertib + Fulvestrant*	Fulvestrant + Placebo	10.3 mo. vs. 4.8 mo.	29.3 mo.vs. 23.4 mo.; HR 0.66	41% vs. 12%	61% vs. 35%	88% diarrhea (14% grade3/4), 62% rash (20% grade 3/4), 45% nausea, 32% hypertension (22% grade 3)	–	–	Supports Capivasertib for PI3K-mut mBC
BEECH (2015)	2	AstraZeneca	2012-2016	ER+ advanced BC	*Capivasertib + Paclitaxel*	Placebo + Paclitaxel	10.9 mo. vs. 8.4 mo., HR 0.80	N/A	39.5% vs. 35.4%	55.3% vs. 49.2%	Diarrhea 76.4% (grade 3 12.7%), anemia 39.3% rash 36% (2% grade 3), fatigue 33%, hyperglycemia 40% (4% grade 3), grade 3/4 neutropenia 7.2%	–	–	Not recommended
INAVO 120 (2024/2025)	3	Genentech	2019-2023	PIK3CA-mutated HR+ mBC	*Inavolisib + Palbociclib + Fulvestrant*	Placebo + Palbociclib + Fulvestrant	17.2 mo. vs. 7.3 mo.	34 mo. vs. 27 mo.	62.7% vs. 28%	75.2% vs. 47%	Grade 3/4 neutropenia 80.2%, grade 3/4 thrombocytopenia 14.2%, grade 3/4 hyperglycemia 5.6%, grade 3/4 stomatitis 5.6%	Oct-24	Not yet	Second line in advanced mBC with PIK3CA mutation
BELLE-3 (2017)	3	Novartis	2013-2016	Postmenopausal women with HR+/HER2- advanced BC after progression on ET or mTORi	*Buparlisib + Fulvestrant*	Placebo + Fulvestrant	3.9 mo. vs. 1.8 mo., HR 0.67	33.2 mo. vs. 31.6 mo., HR 0.92	7.6% vs. 2.1%	25.5% vs. 17.4%	Hyperglycemia 40%, elevated ALT 30%, elevated AST 23%, fatigue 23%, nausea 22%, diarrhea 21%, rash 18%, hypertension 6% (grade 3/4 4%)	no	no	Not recommended
BELLE-2 (2017)	3	Novartis	2012-2014	HR+/HER2- advanced BC	*Buparlisib + Fulvestrant*	Placebo + Fulvestrant	6.9 mo. vs. 5 mo., HR 0.78	N/A	11.8% vs. 3.1%	30.6% vs. 18.4%	Hyperglycemia 39% (grade 3 26%), elevated liver enzymes 28% (grade 3 14%), rash 25% (grade 3 8%), diarrhea 24% (grade 3 2%), fatigue 23% (grade 3 2%), depression 12% (grade 3 2%)	no	no	Not recommended
SANDPIPER (2018)	3	Genentech	2015-2018	HR+/HER2- advanced BC with PIK3CA mutations	*Taselisib + Fulvestrant*	Placebo + Fulvestrant	7.4 mo. vs. 5.4 mo., HR 0.70	N/A	28% vs. 11.9%	51.5% vs. 32.5%	Diarrhea 49% (grade 3 12%), hyperglycemia 40% (grade 3 10%), nausea 22% (grade 3 1%), stomatitis 21% (grade 3 3%), rash 20% (grade 3 2%)	no	no	Not recommended
Antibody Drug Conjugates (ADCs)														
DESTINY-Breast04	3	2022	2018-2021	HR+/HER2-low mBC	*Trastuzumab Deruxtecan (T-DXd)*	CHT	10.1 mo. vs. 5.4 mo. (HR+), HR 0.51	23.9 mo.vs. 17.5 mo. (HR+), HR 0.64	52.6% vs. 16.3% (HR+)	57.5% vs. 30.5%	73% nausea (7.6% grade 3), 47% fatigue (8.4% grade 3), 44% vomiting (6.6% grade 3) 37% alopecia, 34% constipation, 14 neutropenia (12% grade 3), 11% anemia (8.7% grade 3), 12% ILD (0.8% grade 3/4)	Aug-22	Sep-22	HER2-low and HER2-ultralow mBC, after previous CHT or with disease progression during or within 6 mo. of completing adj CHT
DESTINY-Breast06 (2024)	3	Daiichi Sankyo and AstraZeneca	2020-2023	HR+/HER2- low and ultralow mBC	*Trastuzumab Deruxtecan (T-DXd)*	CHT	13.2 mo. vs. 8.1 mo., HR 0.63	23.4 mo. vs. 18.8 mo., HR 0.64	57.3% vs. 31.2%	70.6% vs. 46.1%	73% nausea (7.6% grade 3), 47% fatigue (8.4 grade 3), 44% vomiting (6.6% grade 3), 37% alopecia, 14% neutropenia (12% grade 3/4), 11% anemia, 7% thrombocytopenia	Jan-25	Apr-25	Targeting HER2-ultralow BC
DAISY (2023)	2	Daiichi Sankyo and AstraZeneca	2019-2021	HER2 low/ultralow mBC	*Trastuzumab Deruxtecan*	No comparator	HER2 low 7 mo., HER2 ultralow 4.2 mo.	Not reached	HER2 + 70.6%, HER2 low 37.5%, HER 2 uktralow 29.7%	HER2 + 80.6%, HER2 low 55.4%, HER2 ultra low 50%	25% neutropenia, 10% fatigue, 10% vomiting	Aug-22	Sep-22	HER2-low and HER2-ultralow mBC, after previous CHT or with disease progression during or within 6 mo. of completing adj CHT
TROPION-Breast01 (2023)	3	Daiichi Sankyo and AstraZeneca	2021-2023	HR+/HER2- mBC	*Datopotamab Deruxtecan*	Standard CHT	6.9 mo. vs. 4.9 mo., HR o.63	Not significant	36.4% vs. 22.9%	50% vs. 42%	56% stomatitis, 40% keratitis, 3% ILD	Jan-25	Apr-25	Second-line therapy unresectable/HR+ mBC who have previously received ET and CHT in the metastatic setting
TROPICS-02 (2023)	3	Gilead	2019-2021	HR+/HER2- mBC, heavily pretreated	*Sacituzumab Govitecan (SG)*	CHT	5.5 mo. vs. 4 mo., HR 0.66	14.4 mo.vs. 11.2 mo., HR 0.79	21% vs. 14%	34% vs. 22%	54% neutropenia (38% grade 3/4, 51% diarrhea (9% grade 3/4), 38% anemia (7% grade 3/4), 34% fatigue, 33% nausea, 25% vomiting, 24% leukopenia (3% grade 3/4), 6% febrile neutropenia (3% grade 3/4)	Apr-23	Jun-23	HR+/HER2- mBC, after ET and at least 2 previous lines of therapy for mBC

BC, Breast Cancer; HR+, Hormone Receptor-Positive; HER2-, Human Epidermal Growth Factor Receptor 2-Negative; mBC, Metastatic Breast Cancer; pts., patients; PFS, Progression-Free Survival; OS, Overall Survival; ORR, Overall Response Rate; CBR, Clinical Benefit Rate; FDA, Food and Drug Administration; EMA, European Medicines Agency; SERMs, Selective Estrogen Receptor Modulators; OFS, Ovarian Function Suppression; ET, Endocrine Therapy; AIs, Aromatase Inhibitors; SERDs, Selective Estrogen Receptor Degraders; CDK4/6i, Cyclin-Dependent Kinase 4/6 Inhibitors; mTORi, Mechanistic Target of Rapamycin Inhibitors; PARPi, Poly(ADP-ribose) Polymerase Inhibitors; PIK3CAi/AKTi, Phosphatidylinositol 3-Kinase Inhibitors and AKT Inhibitors; ADCs, Antibody Drug Conjugates; CHT, Chemotherapy.

In HR+ mBC, ET is better tolerated than CHT, and better tumor control is achieved with ET than with CHT. According to current ESMO guidelines, the preferred first-line approach in the metastatic setting is ET in combination therapy with CDK4/6i. More recently, this has also become standard in early-stage high risk BC patients (23). While tamoxifen remains a viable option for premenopausal patients with childbearing potential, non-steroidal AIs such as anastrozole and letrozole are generally preferred due to their superior effectiveness in achieving disease control. Moreover, they are even more potent when combined with CDK4/6i, although they seem to be directly related to the development of estrogen receptor 1 (ESR1) mutations.

In case of disease progression, several treatment options should be considered based on the presence of targetable mutations, including ESR1. To overcome this resistance mechanism, newer selective estrogen receptor degraders (SERD) such as elacestrant (24) and other novel antiestrogens such as complete estrogen receptor antagonists (CERANs), selective estrogen receptor covalent antagonists (SERCAs), and proteolysis targeting chimera (PROTACs) have been evaluated in clinical studies (25, 26).

**Figure 1:**
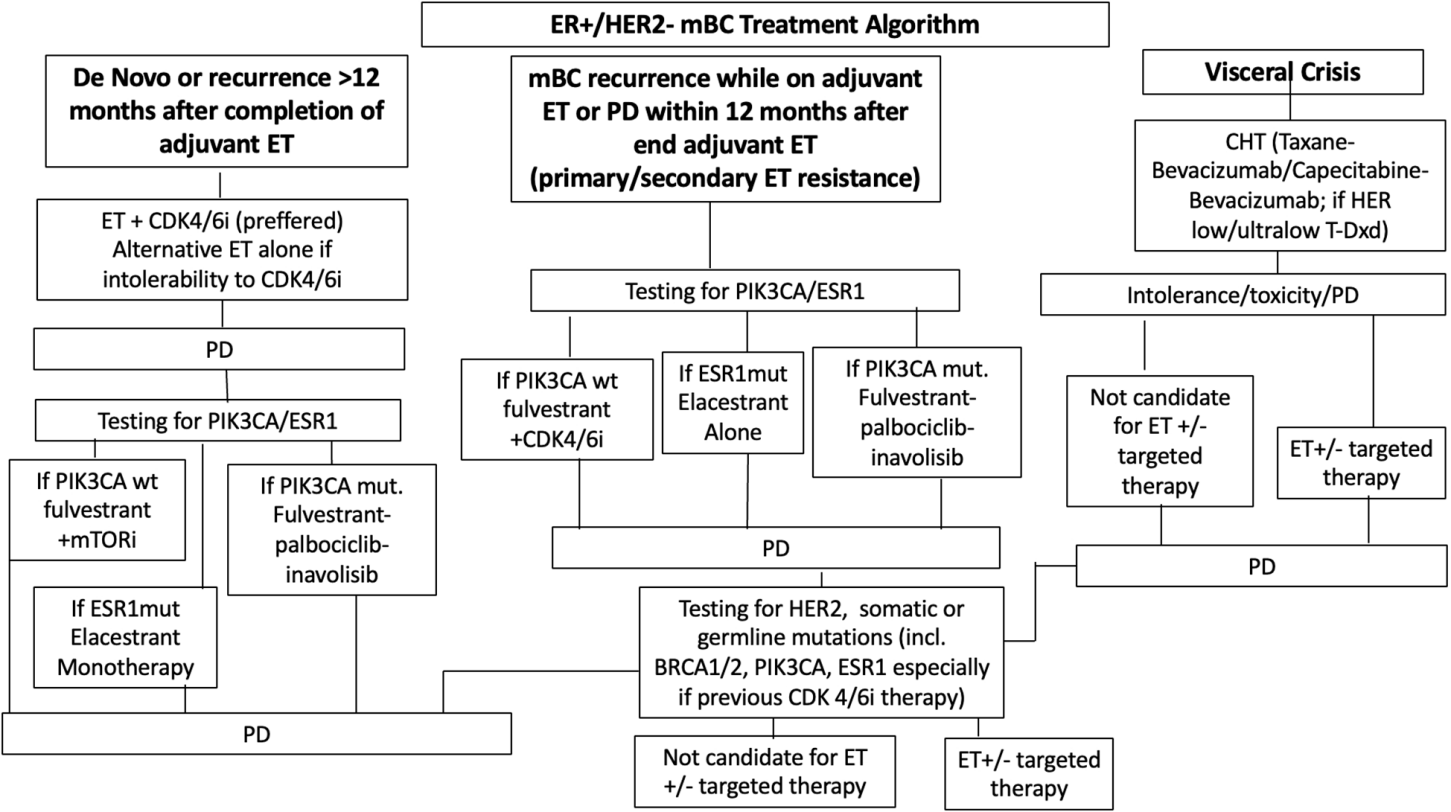
Treatment paradigm for hormone receptor positive (HR+) metastatic breast cancer. ET, endocrine therapy; CDK4/6i, cyclin-dependent kinase 4/6 inhibitor; AI, aromatase inhibitor; CHT, chemotherapy; PD, progressive diseases.

In those cases where targeted treatment is not available, combination with aromatase inhibitors and everolimus still remain an option (27) (ESMO Metastatic Breast Cancer Living Guidelines, 2023) (**Figure 1**).

For women with rapidly progressing disease or visceral crisis, CHT continues to be the preferred strategy, with anthracyclines and taxanes being the most used agents (28). However, recent advances have delivered more modern CHT options, such as antibody-drug conjugates (ADCs). Trastuzumab deruxtecan (T-DXd) has emerged as a key agent for patients with HER2-low disease (defined as IHC 1+ or 2+ with ISH <2), while sacituzumab govitecan is playing an increasingly important role in later-line settings (27) (**Figure 1**).

Beyond tumor-specific risk factors, therapy selection is becoming increasingly personalized taking into account factors such as age, menopausal status, performance status, comorbidities, and prior treatment history. Emerging combination and sequencing strategies are gaining attention, aiming to de-escalate CHT while extending progression-free survival (PFS).

Supporting this approach, recent findings from the RIGHT Choice trial showed that first-line ribociclib plus ET improved median PFS when compared with combination CHT, had comparable overall response rates (ORR) and lower rates of adverse events in HR+/HER2– advanced BC (29).

Main treatment paradigm for HR+ mBC is illustrated in **Figure 1**.

## Pharmacologic landscape of HR+/HER2− mBC

### Aromatase inhibitors

Aromatase inhibitors (AIs) are designed to block the activity of CYP19A1, an aromatase enzyme belonging to the cytochrome P450 family, which catalyzes the conversion of androgens to estrogens in peripheral tissues. These tissues—such as adipose tissue, skin, and muscle—lie outside the gonads and become the primary sites of estrogen synthesis in postmenopausal women. By suppressing this peripheral conversion, AIs effectively lower circulating levels of estradiol and limit estrogen-driven tumor growth.

Among them, third-generation non-steroidal AIs (letrozole and anastrozole) are commonly used in combination with CDK4/6i (30) in first-line treatment for HR+ mBC. In later treatment lines, the steroidal AI exemestane is often paired with the mTOR inhibitor everolimus (24), particularly in the absence of visceral crisis or imminent organ failure that would necessitate chemotherapy.

**Figure 2:**
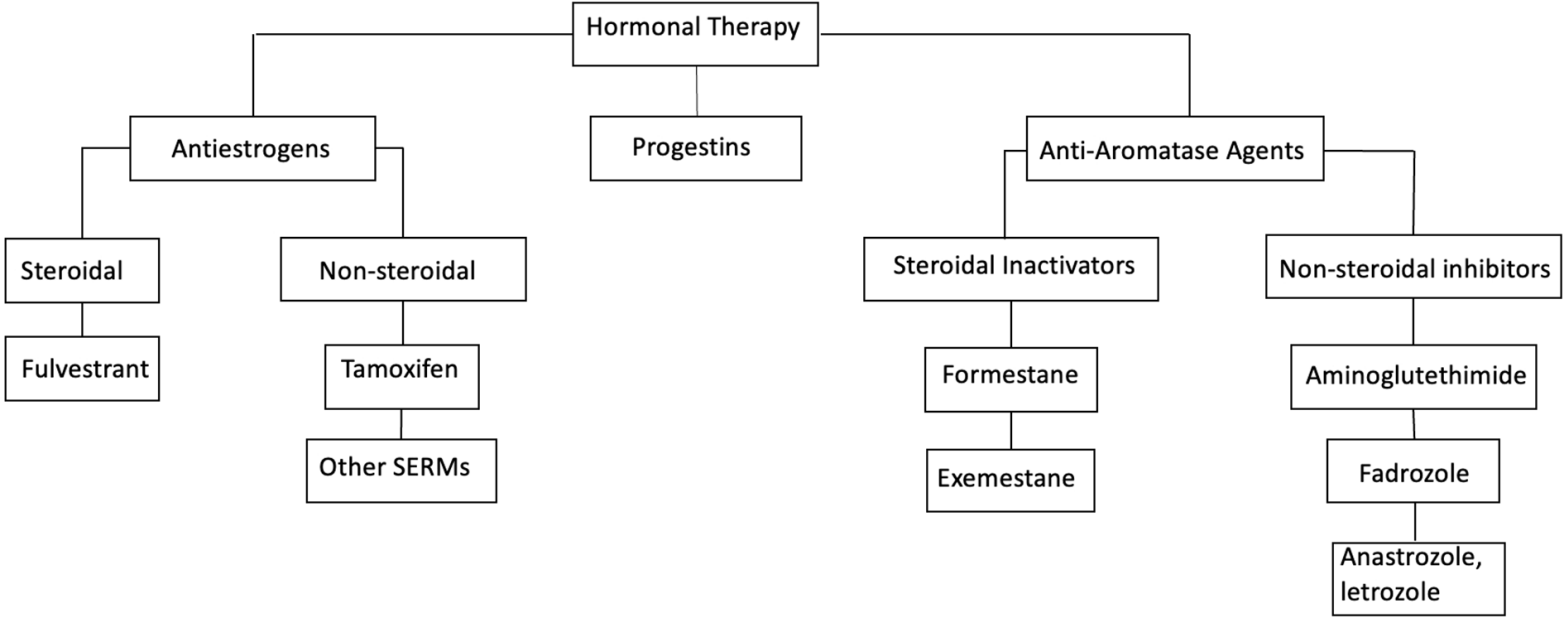
Classification of hormonal therapy agents. SERM, selective estrogen receptor modulators.

Classification of common hormonal agents is illustrated in **Figure 2**.

Significant structural differences allow for different mechanism of actions among steroidal and non-steroidal AIs, making these drugs lack cross-resistance, and supporting their individual use in different circumstances. As such, exemestane binds to the androgen substrate anchoring site of the aromatase enzyme and permanently inactivates it, while non-steroidal AIs block the peripheral conversion of androstenedione to estrone through reversible covalent bonding with the heme moiety of the aromatase – see **Table 2**. All third-generation AIs inhibit about 97-99% of peripheral aromatization (31).

**Table 2:** Classification and comparison of steroidal *vs*. non-steroidal aromatase inhibitors (AIs, their mechanism of action, resistance and side effects).

AIs	Steroidal	Non-Steroidal
**Examples**	**Exemestane**	**Anastrozole, Letrozole**
**Mechanism of action**	**Irreversibly** bind to the active site of the aromatase enzyme, specifically at the substrate-binding pocket	**Reversibly** bind to the **heme group** of the aromatase enzyme’s active site, blocking iron atoms from catalyzing the conversion of androgens (androstenedione or testosterone) into estrogens
**Binding**	Covalent (“suicidal inhibition”)	Non-covalent
**Risk of resistance**	Lower	Higher
Side Effects		
Hot Flashes	Common	Common
Arthralgia	Common	More frequent
Bone Density Loss	Less severe	More pronounced
Androgenic Effects	Possible	None
Cardiovascular Risk	Minimal	Slight increase (due to cholesterol changes)
Cognitive Effects	Mild	More common
Mood Changes	Less frequent	More frequent

AI, aromatase inhibitors.

**Figure 3:**
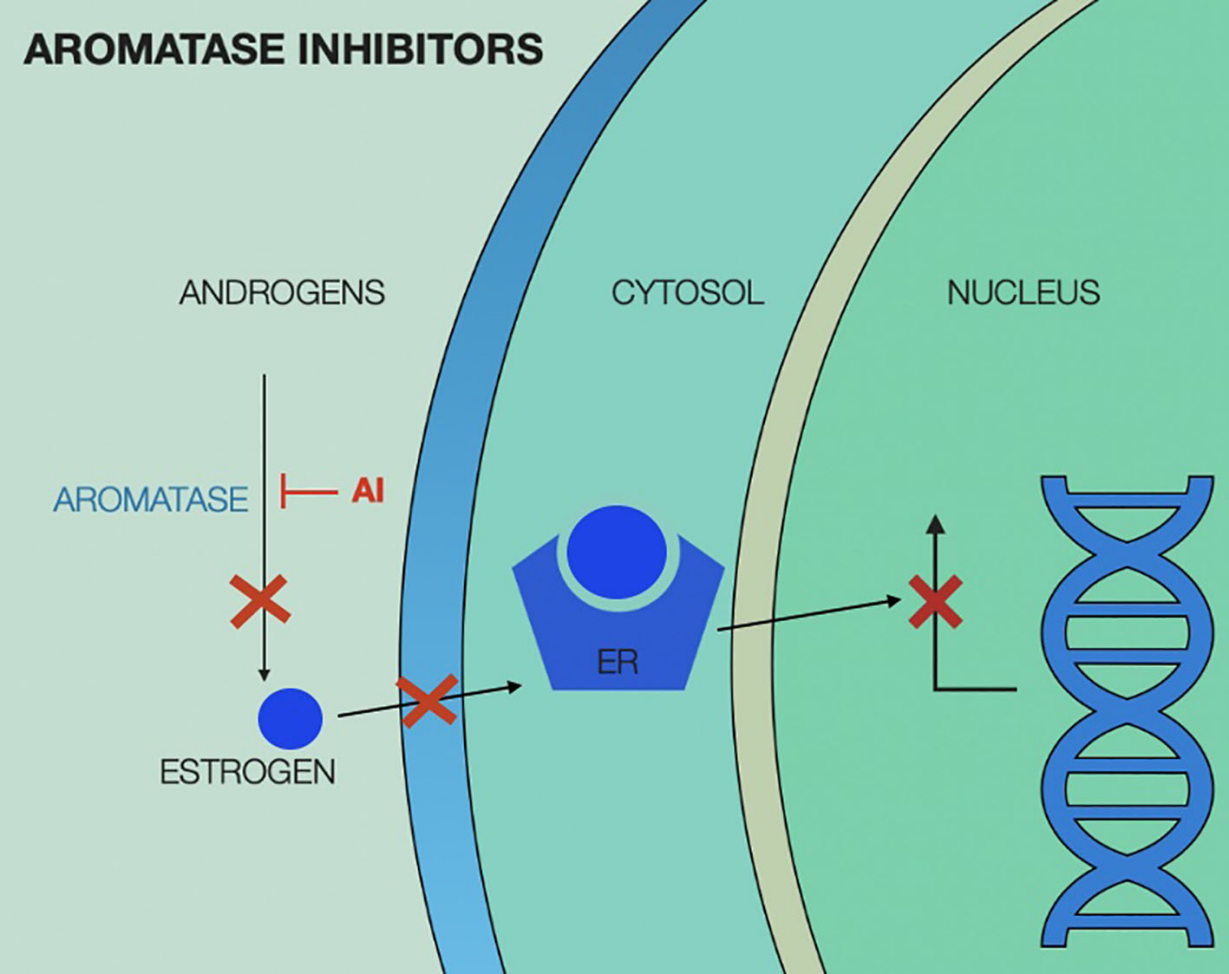
Aromatase inhibitors (AI) mechanism of action, showing interruption of estrogen production in the peripheral tissue, leading to no estrogen translocating in the cytosol and therefore lack of estrogen-estrogen receptor complex to activate nuclear transcription factors. ER, estrogen receptor; AI, aromatase inhibitor.

Mechanism of action of aromatase inhibitors is illustrated in **Figure 3**.

However, while AI monotherapy can achieve a median PFS of 1 to 4 years, when used in first line treatment for mBC, its efficacy declines significantly in later lines, with median PFS dropping to approximately 2 to 6 months in the second-line setting. In some cases, this seems to be due to resistance to AIs, linked to mutations of the ligand-binding domain of the ESR1 (32), which were first discovered in 1997. These mutations result in a constitutively active estrogen receptor that can signal independently of estrogen, rendering AI-induced estrogen depletion ineffective (33). The importance of ESR1 mutations in mBC development has not been discovered until 2013, when genomic sequencing of circulating tumor cells (CTC) in patients with metastatic disease allowed for its identification as a key factor of ET resistance (34–36).

According to current guidelines, endocrine resistance is classified as either primary or secondary. Primary endocrine resistance refers to relapse during the first 2 years of adjuvant ET or progression within the first 6 months of first-line ET for mBC, in the absence of prior ET in the metastatic setting. Secondary resistance is defined as relapse after 2 years of adjuvant therapy or within one year after completion of ET for early BC or progression after 6 months of ET in the metastatic setting (5).

ET resistant mutations, such as ESR1 are usually found in patients with secondary endocrine resistance (27).

In patients who received AI in the neoadjuvant setting, ESR1 mutations are found in 1.5%-7%, whereas in those who received AI for recurrent BC after previous adjuvant ET, it ranges from 4% to 5%. The highest prevalence of ESR1 mutations was documented in patients who have previously received AI for mBC, where it was found to range between 20%-40%, while in ET naïve mBC patients, ESR1 mutations were present in 1% of cases, suggesting that these mutations are primarily induced by AIs (32). Moreover, resistance is influenced by the choice of drug, with a specific 30-fold reduction in binding affinity for tamoxifen and a 40-fold reduction for fulvestrant, necessitating higher dosages. Resistance also appears to be driven by specific ESR1 mutations, with the Y537S mutation associated with a worse prognosis compared to D538G (37).

In metastatic breast tumors harboring ESR1 mutations, selective estrogen receptor modulators (SERMs) and selective estrogen receptor covalent antagonists (SERCAs) seem to work better than AI.

With regards to combination therapies, in addition to CDK4/6 inhibitors, tyrosine kinase inhibitors (TKIs) have been studied in combination with AIs for HR+ mBC. While clinical trials have shown some benefit, their use is currently limited to HER2-positive disease (38).

### Selective estrogen receptor modulators

Tamoxifen is the most extensively evaluated SERM, with antagonistic effects in the breast but predominantly agonistic effects in the endometrial and liver tissue. Tamoxifen binds to the ER, leads to homodimerization of the complex, and translocation to the nucleus, where it blocks binding of co-activators and promotes binding of co-repressors, blocking transcription of activation factor 2 domain (AF2), but not of AF1. This explains its partial agonistic effects in the uterus (39). Tamoxifen has been a cornerstone of treatment in mBC since the 1970s, following the discovery of its ability to stop tumor growth, preserve bone density, and lower serum cholesterol. Long-term follow-up from early clinical trials demonstrated improved 15-year outcomes, with average annual reductions in the risk of relapse and mortality by 12% and 9%, respectively (11). In metastatic disease the median duration of response to tamoxifen therapy is 9–12 months, with an overall response rate of 30–40%. Response rates are even higher in patients with soft tissue metastases (35%) compared to those with visceral (29%) or bone metastases (25%) (11).

Notably, even in early stages HR+ BC, ET with tamoxifen or AI for 5–10 years were until recently, standard treatment. Front-line therapy with AIs has demonstrated a 3.6% reduction in 10-year recurrence risk and a 2.1% improvement in OS compared to tamoxifen. However, AIs are particularly advantageous for patients with advanced-stage (node-positive), high-grade, HER2-positive, or highly proliferative BC and are the preferred choice for lobular cancers. As a result, an upfront AI or a sequential approach—starting with tamoxifen for 2–5 years followed by an AI—were long considered the standard of care, achieving a 2% reduction in recurrence risk and a 1.5% decrease in risk of mortality when compared to tamoxifen alone (40).

In premenopausal women with stage I–III BC, the ASTRRA trial showed that addition of ovarian function suppression (OFS) to tamoxifen led to a 5-year PFS rate of 91.1%, compared to 87.5% with tamoxifen alone (HR 0.69, 95% CI 0.48–0.97, p=0.033). This benefit was sustained at the 8-year follow-up, when PFS rate reached 85.4% in the tamoxifen plus OFS group versus 80.2% in the tamoxifen monotherapy group (HR 0.67, 95% CI 0.51–0.87) (41). Despite these findings, no significant OS benefit was observed (96.5% in the OFS group *vs*. 95.3% in the tamoxifen group, HR 0.78, 95% CI 0.49–1.25) (42).

Common side effects of tamoxifen include hot flashes and vaginal bleeding, but more serious side effects, such as thromboembolic events (3–4%), must also be considered. Despite its well-established benefits, tamoxifen use is associated with a significantly elevated risk of endometrial cancer, with reported incidence rates of 2.20 per 1000 woman-years, compared to 0.71 per 1000 woman-years observed with placebo (28).

Raloxifene, a SERM with a favorable risk profile and lacking the agonistic effects of tamoxifen, demonstrated no significant benefit in a heavily pre-treated population. However, a clinical benefit rate of 33% was observed when combining partial responses with prolonged stable disease in 21 patients with HR+ mBC (43). Currently, raloxifene has limited applications in the mBC setting.

**Figure 4:**
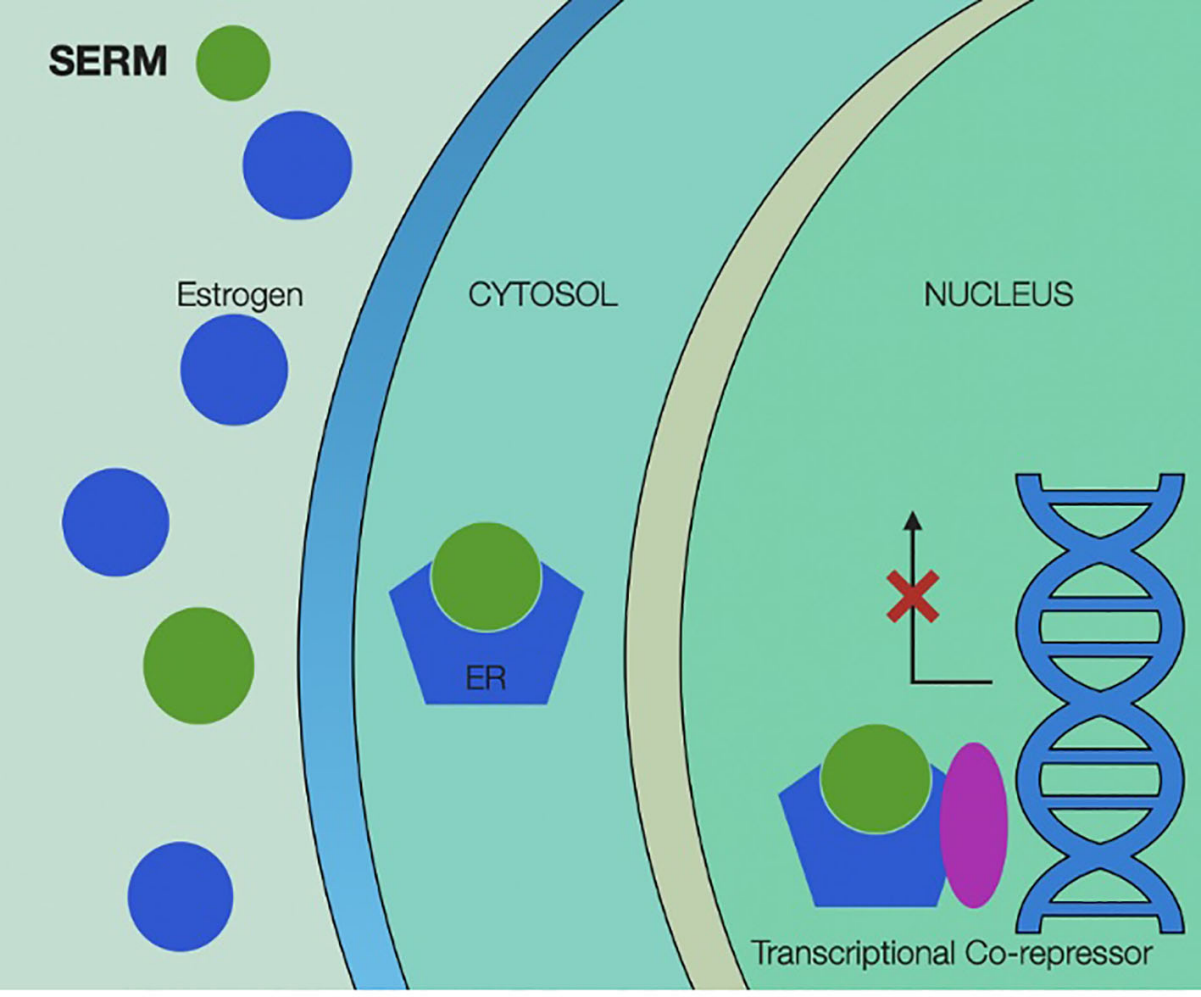
Selective estrogen receptor modulators’ mechanism of action, showing binding of the selective modulator by estrogen receptor and activating transcriptional co-repressors inside the cancer cell’s nucleus. SERM, selective estrogen receptor modulators; ER, estrogen receptor.

Mechanisms of action of SERMs are illustrated in **Figure 4**.

Among newer SERMs, the oral agent lasofoxifene has been evaluated in the phase II ELAINE 1 trial in ESR1-mutated, endocrine therapy–resistant mBC following progression on AI plus CDK4/6 inhibitors. While differences in PFS and response rates compared to fulvestrant did not reach statistical significance, lasofoxifene was associated with a more pronounced reduction in circulating ESR1 mutant allele fraction (82.9% drop from baseline at week 8 compared to 61.5% in the fulvestrant arm), suggesting potential biological activity warranting further investigation (44).

### Selective estrogen receptor degraders

Fulvestrant is a SERD approved for the treatment of HR+ BC following progression on SERM and AI. It binds the ligand-binding domain of ERα, inhibiting conformational changes at both AF-1 and AF-2, and preventing co-activators recruitment. Because the complex is unstable, it degrades, leading to pure ER antagonism. Intramuscular injection of fulvestrant every 4 weeks reduces ER expression in a dose-depending fashion. Common side effects are menopause-like symptoms (28).

Benefit of fulvestrant was established at the beginning of the century when it was directly compared to AI in treatment of postmenopausal women with mBC or locally advanced BC progressing after ET (45, 46). Subsequent trials, such as FIRST and FALCON, demonstrated the benefits of a higher fulvestrant dose (500 mg versus 250 mg) with a loading dose strategy as first-line treatment in ET-naïve mBC, showing superiority over AIs (47). Loading dose strategies were later indirectly evaluated in two clinical trials, along with possible additional combinations – SoFEA (48) and EFFECT (48). Notably, the SoFEA trial found no PFS benefit with the addition of anastrozole to fulvestrant compared to fulvestrant alone (4.4 months [95% CI 3.4–5.4] *vs*. 4.8 months [3.6–5.5], respectively) or exemestane monotherapy (3.4 months [3.0–4.6]) (49). However, both trials confirmed efficacy of fulvestrant modality of application with 500 mg intramuscularly on day 0, 250 mg on days 14, 28, and 250 mg every 28 days thereafter.

This was later reinforced through the CONFIRM trial, which showed that fulvestrant 500 mg led to a significant improvement in median OS to 26.4 months compared to 22.3 months with the 250 mg dose (HR, 0.81; 95% CI, 0.69–0.96; p=0.02) (50). It is important to note that these studies included patients who had progressed on prior non-steroidal AIs and had acquired endocrine resistance. In contrast, the S0226 evaluated fulvestrant in ET–naïve postmenopausal patients with mBC and showed improved outcomes with the combination of fulvestrant and anastrozole. In this population, median PFS was 15.0 months with combination therapy compared to 13.5 months with anastrozole alone (HR 0.81, p = 0.007), and extended to 16.7 *vs*. 12.7 months in patients who were entirely endocrine-naïve. Overall survival was also prolonged (49.8 *vs*. 42.0 months; HR 0.82, p = 0.03), with the most pronounced benefit observed in tamoxifen-naïve patients (52.2 *vs*. 40.3 months; HR 0.73). No significant OS advantage was seen in those previously treated with tamoxifen (51).

In ESR1-mutated BC a combined analysis of the SoFEA and EFECT trials further demonstrated higher OS at one year with fulvestrant (80%) *vs*. exemestane (62%) (p=0.04). In contrast, among patients without ESR1 mutations, 1-year OS was similar between the two treatments (81% for fulvestrant *vs*. 79% for exemestane, p=0.69). Additionally, the EFECT trial reported no significant difference in response rate or clinical benefit between fulvestrant and exemestane in an unselected metastatic cohort. These findings suggest a survival advantage for fulvestrant in ESR1-mutated disease, while both agents appear equally effective in wild-type cases (51, 52). More recently, the Chinese FRIEND trial further supported the superiority of fulvestrant over exemestane, demonstrating improved PFS (8.5 *vs*. 5.6 months, p=0.014), ORR (19.5% *vs*. 6%, p=0.017), and time to treatment failure (8.4 *vs*. 5.5 months, p=0.008). However, in this trial, no significant difference in efficacy was observed between ESR1-mutated and ESR1 wild-type HR+ mBC (53).

Fulvestrant-based combination therapies, especially with CDK4/6i, have been evaluated in multiple trials, which will be explored in the following sections.

To address the limitations of fulvestrant and provide improved bioavailability and efficacy compared to AIs, particularly in somatic ESR1-mutated BC, novel oral SERDs are currently under development. Among these, elacestrant has shown promising PFS results (54) and it is approved for clinical use since 2023.

Elacestrant is a nonsteroidal oral SERD that degrades ER-alpha in a proteasome dose-dependent manner. It also inhibits estradiol-dependent, ER-directed gene transcription, thereby suppressing tumor growth in heavily pretreated BC, including both non-mutated and ESR1-mutated cases (55).

Recent findings from the EMERALD trial have demonstrated the efficacy of elacestrant in HR+ mBC, particularly within the ESR1 mutated breast tumors. The trial reported improved 1-year PFS in all patients (HR 0.70, 95% CI 0.55–0.88, p=0.002), including those with ESR1-mutated tumors (HR 0.55, 95% CI 0.39–0.77, p=0.005), without significant limiting side (54) effects (54).

Additionally, the SERENA-2 trial demonstrated the efficacy of camizestrant, a next-generation selective estrogen receptor degrader (ngSERD), in patients with advanced HR+ HER2- BC who had progressed or relapsed after ≤1 line of ET and ≤1 line of CHT in the advanced setting, without prior exposure to fulvestrant or other SERDs. The primary endpoint was PFS, comparing camizestrant at 75 mg and 150 mg daily to fulvestrant 500 mg. Median PFS was 7.2 months (90% CI, 3.7–10.9) with camizestrant 75 mg, 7.7 months (90% CI, 5.5–12.9) with 150 mg, and 3.7 months (90% CI, 2.0–6.0) with fulvestrant. Both camizestrant doses significantly reduced the risk of progression versus fulvestrant, with HRs of 0.59 (90% CI, 0.42–0.82; p=0.017) for 75 mg and 0.64 (90% CI, 0.46–0.89; p=0.009) for 150 mg (56).

Patients previously treated with CDK4/6 inhibitors had poorer outcomes, with PFS of 5.5 months (HR 0.49) in the camizestrant 75 mg plus fulvestrant group, 3.8 months (HR 0.68) with 150 mg, and 2.1 months with fulvestrant alone. Among patients with ESR1 mutations, PFS was 6.3 months (HR 0.33) with 75 mg of camizestrant, 9.2 months (HR 0.655) with 150 mg, and 2.2 months with fulvestrant (56, 57).

Most recently, the SERENA-6 phase III trial, presented at ASCO 2025, demonstrated that camizestrant, when administered at 75 mg daily in combination with a CDK4/6 inhibitor to patients with detectable emergent ESR1 mutations during ongoing AI plus CDK4/6i therapy, significantly improved outcomes. The median PFS was 16.0 months with camizestrant versus 9.2 months with continued AI (HR 0.44; 95% CI, 0.31–0.60; p<0.00001). Camizestrant also substantially prolonged time to global health status deterioration (23.0 *vs*. 6.4 months; HR 0.53; 95% CI, 0.33–0.82; p<0.001) (58), positioning it as a promising early intervention strategy upon molecular progression. Notably, SERENA-6 is the first global registrational study to validate ctDNA-guided treatment intensification based on ESR1 mutation emergence, highlighting its potential to redefine endocrine sequencing.

More recently, results from the phase III EMBER-3 trial were also published. This study investigated the oral SERD imlunestrant in patients with ER-positive, HER2-negative mBC who progressed on prior AI therapy, with or without CDK4/6 inhibitors. Patients were randomized to imlunestrant, standard ET, or imlunestrant plus abemaciclib. In the ESR1-mutant subgroup, imlunestrant significantly improved PFS compared to standard ET (5.5 *vs*. 3.8 months; P<0.001). In the overall population, PFS was similar between imlunestrant and standard ET (5.6 *vs*. 5.5 months; HR 0.87; P=0.12). However, the combination of imlunestrant with abemaciclib yielded superior PFS (9.4 *vs*. 5.5 months; HR 0.57; P<0.001), regardless of ESR1 status. Imlunestrant alone was well tolerated, with fewer grade ≥3 adverse events than the combination arm (59). These findings support imlunestrant as a promising endocrine backbone, particularly in ESR1-mutant mBC.

Other SERDs such as giradestrant and amcenestrant, evaluated in the acelERA and AMEERA-3 studies respectively, showed no benefit over traditional ET (60), despite previous promising findings. Giradestrant was initially evaluated as monotherapy (30 mg orally daily) versus combination therapy with palbociclib in postmenopausal women with HR+ mBC, demonstrating an ORR of 38% in the combination arm compared to 20% in the monotherapy arm. Paired biopsies from 21 patients taken before and during treatment revealed downregulation of ER, PR, Ki67, and ER pathway activity, along with a significant reduction in ESR1 ctDNA mutations detected in 94% of patients after 4 weeks of therapy (26).

Combination trials involving giradestrant and palbociclib versus letrozole and palbociclib, as well as giradestrant and everolimus versus exemestane and everolimus, are currently ongoing. Additionally, a randomized umbrella trial is evaluating giradestrant in various combination with ipatasertib, inavolisib, everolimus, and samuraciclib (26).

**Figure 5:**
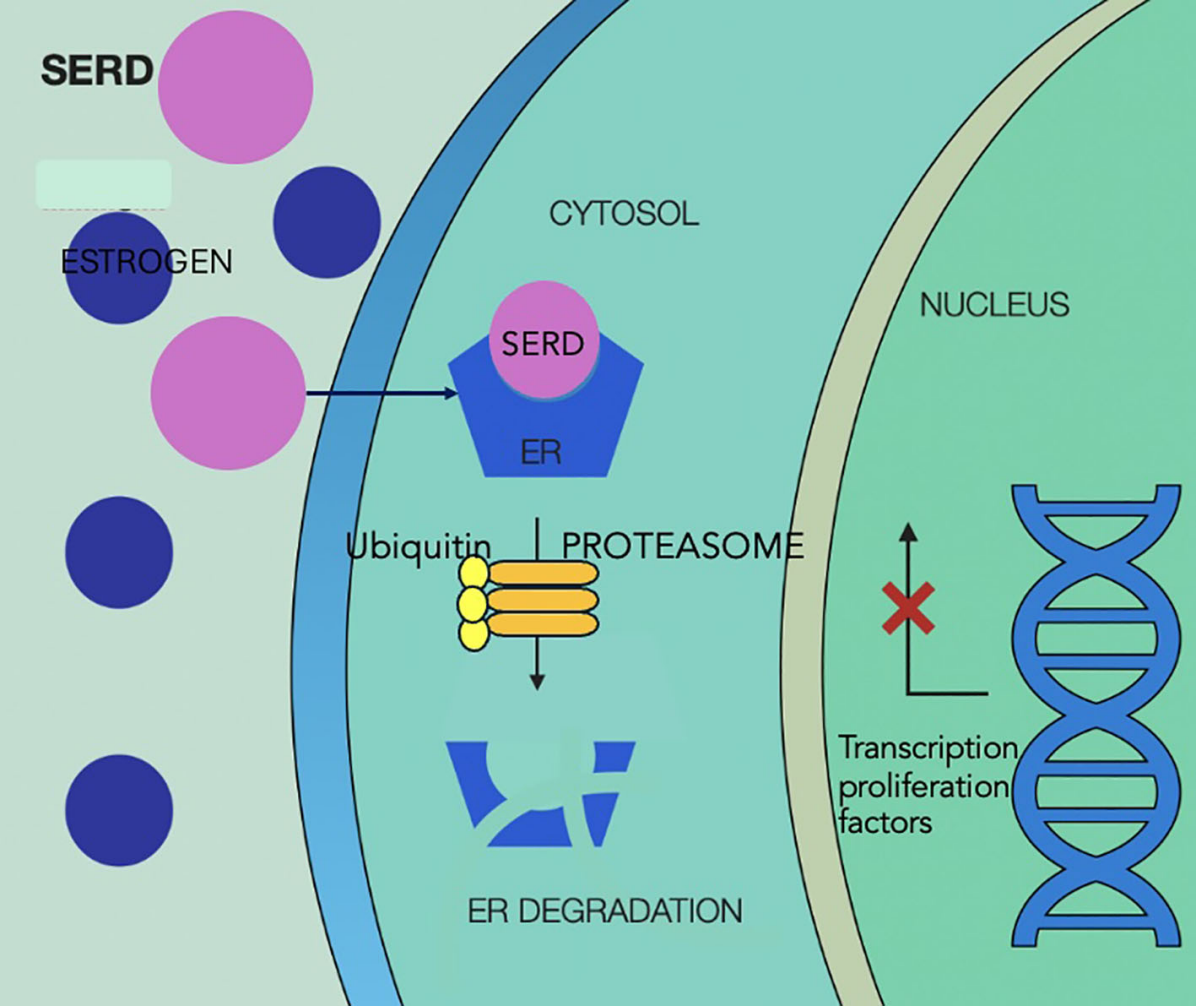
Selective estrogen receptor degraders mechanism of action, showing competitive binding of SERD to the estrogen receptor in the cytoplasm, displacing estrogen and preventing receptor activation. SERD binding leads to conformational change in the receptor, making it a target for ubiquitin-mediated degradation via the proteasome pathway, reducing levels of estrogen receptor and estrogen-driven gene expression in the nucleus, which ultimately leads to less activation of transcription factors and inhibition of tumor proliferation. SERD, selective estrogen receptor degraders; ER, estrogen receptor.

Mechanisms of action of SERDs are illustrated in **Figure 5**.

### CDK4/6 inhibitors

The introduction of CDK4/6 inhibitors has been one of the most transformative advances in the treatment of HR+/HER2– mBC over the past decade, leading to significant survival benefits. Palbociclib, ribociclib, and abemaciclib—routinely used in combination with ET—now represent the standard first-line approach for most patients with HR+/HER2– mBC (61).

CDK4/6 forms active complexes with D-type cyclins, driving hyperphosphorylation of the retinoblastoma (Rb) protein. This inactivates Rb, leading to the release of E2F transcription factors that promote cell cycle progression from G1 to S phase. Dysregulation of this pathway is common in cancer, facilitating uncontrolled cellular proliferation (61). Estrogen induces expression of cyclin D and promotes CDK4/6 activity in HR+ BC. Therefore, estrogen depletion is essential for cell-cycle arrest.

CDK4/6 inhibitors are currently approved in combination with ET for HR+ mBC, with abemaciclib additionally approved as monotherapy.

For endocrine-sensitive cases, AI-based combinations were evaluated in PALOMA-2, MONALEESA-2, and MONARCH-3, reporting hazard ratios for progression of 0.54, 0.55, and 0.58, respectively, with median PFS gains of 9–13 months. Specifically, median PFS was 24.8 months with palbociclib plus letrozole compared to 14.5 months with placebo plus letrozole in PALOMA-2 (62), and 25.3 months with ribociclib plus letrozole versus 16 months with placebo plus (61) letrozole in the MONALEESA-2 study (63). Abemaciclib also showed significant benefit, achieving a PFS of 28.18 months in combination with anastrozole/letrozole *vs*. 14.76 months in the placebo plus anastrozole/letrozole group (64).

Regarding OS ribociclib is the only CDK4/6 inhibitor to show a statistically significant OS benefit in the first-line setting. In the MONALEESA-2 trial, ribociclib plus letrozole achieved a median OS of 63.9 months (95% CI, 52.4–71.0) versus 51.4 months (95% CI, 47.2–59.7) with placebo plus letrozole (HR 0.76; 95% CI, 0.63–0.93; p=0.008) (65). In contrast, the PALOMA-2 trial failed to demonstrate a significant OS advantage for palbociclib plus letrozole compared to placebo (53.9 *vs*. 51.2 months; HR 0.956; p=0.3378) (62). Similarly, the MONARCH-3 trial, with 8 years of follow-up, reported a non-significant OS improvement of 13.1 months for abemaciclib plus AI (66.8 *vs*. 53.7 months; HR 0.804; p=0.0664) (66).

In *endocrine-resistant disease*, CDK4/6i were investigated in combinations with fulvestrant-based ET. Importantly, primary endocrine resistance does not preclude clinical benefit from subsequent endocrine-based combinations. Several studies, including PALOMA-3, MONARCH 2 and MONALEESA-3, have demonstrated that CDK4/6 inhibitors such as palbociclib, abemaciclib and ribociclib, when combined with fulvestrant, retain efficacy in both primary and secondary endocrine-resistant HR+/HER2− mBC.

In PALOMA-3, the combination of palbociclib and fulvestrant significantly improved PFS compared to fulvestrant plus placebo in HR+/HER2– mBC patients who had progressed on prior ET. Median PFS was 9.5 months versus 4.6 months (HR 0.46; p<0.0001), and objective response rate (ORR) was notably higher with palbociclib (24.6% *vs*. 10.9%). Importantly, this benefit was independent of PIK3CA mutation status (67).

Similarly, MONARCH-2 evaluated abemaciclib in combination with fulvestrant in a chemotherapy-naïve population with progression on prior ET. Abemaciclib significantly prolonged PFS to 16.4 months compared to 9.3 months with placebo (HR 0.553; p<0.001), with an ORR of 48.1% versus 21.3%, respectively — representing a gain of over 7 months and more than doubling response rates (68).

In MONALEESA-3, ribociclib was assessed alongside fulvestrant in a broader population that included both treatment-naïve and previously treated patients. The combination achieved a median PFS of 20.5 months versus 12.8 months with placebo (HR 0.593; p<0.001). This benefit was consistent across subgroups, including treatment-naïve patients (HR 0.577) and those with prior ET for mBC (HR 0.565). ORR was also higher with ribociclib (40.9%) compared to placebo (28.7%) (69).

Collectively, these findings established all three CDK4/6 inhibitors—palbociclib, abemaciclib, and ribociclib—as key components of ET-based therapy for both endocrine sensitive and endocrine resistant HR+/HER2– mBC.

The clinical benefit of CDK4/6 inhibitors extends beyond postmenopausal, endocrine-sensitive and -resistant settings. In pre- and perimenopausal patients with HR+/HER2– mBC, the combination of ribociclib with a non-steroidal AI and ovarian suppression (goserelin) has also demonstrated significant efficacy. In this population, the MONALEESA-7 trial reported a median OS of 58.7 months with ribociclib versus 48.0 months with placebo (HR 0.76; 95% CI, 0.61–0.96), with 4-year OS rates of 60% and 50%, respectively (70). PFS similarly improved from 13.0 to 23.8 months (HR 0.55; p<0.0001) (71).

Additional evidence supporting the use of CDK4/6 inhibitors in younger patients comes from the Young PEARL trial, a phase II randomized study comparing palbociclib plus ET and ovarian suppression to capecitabine in premenopausal women with HR+/HER2– mBC. Median PFS was 19.5 months (90% CI, 14.3–22.2) in the palbociclib arm versus 14.0 months (90% CI, 11.7–18.7) in the capecitabine arm (HR 0.74; 90% CI, 0.57–0.98; p =0.036) (72), confirming the efficacy of CDK4/6i in this subgroup with a more favorable toxicity profile.

The ABIGAIL trial further explored the role of abemaciclib in premenopausal mBC patients with visceral disease. Patients were randomized to receive either abemaciclib plus ET (letrozole or fulvestrant) or an induction regimen of paclitaxel followed by abemaciclib plus ET. The primary endpoint was met, with a 12-week objective response rate (ORR) of 59% in the abemaciclib plus ET arm compared to 40% in the chemotherapy-sequenced arm (OR 2.12; 95% CI, 1.13–3.96; p=0.019) (73), supporting the efficacy of endocrine-based regimens even in the presence of aggressive disease biology.

Despite the synergy observed with ET combinations, abemaciclib remains the only CDK4/6 inhibitor approved as monotherapy. This indication was based on the MONARCH 1 trial, which evaluated abemaciclib in heavily pretreated, CDK4/6i-naïve patients with HR+/HER2– mBC (65). This trial reported an ORR of 19.7% and a median PFS of 6 months (74). Notably, all participants in this study were CDK4/6i-naïve (61). Subsequent data suggest that abemaciclib retains efficacy even after prior CDK4/6i exposure: in a multicenter cohort, patients previously treated with palbociclib and ET achieved a median OS of 17.2 months and PFS of 5.3 months with abemaciclib, administered either alone or in combination with ET (75).

As briefly mentioned earlier, CDK4/6i were also validated against CHT in the phase II RIGHT Choice trial, which compared ribociclib plus ET to physician’s choice of CHT in 223 patients with HR+ mBC experiencing rapid disease progression. With a median follow-up of 24.1 months, the trial demonstrated nearly a 12-month advantage in median PFS for the ribociclib arm compared to CHT (24 months *vs*. 12.3 months; HR 0.54; 95% CI, 0.36–0.79; p=0.007). ORR were comparable between the groups, with 65.2% for ribociclib and 60% for CHT, although ribociclib showed a slightly longer time to response (29).

These findings are consistent with an earlier phase II study in premenopausal HR+ mBC patients who relapsed or progressed on tamoxifen therapy. This trial compared palbociclib plus ET to capecitabine and demonstrated a median PFS of 20.1 months in the palbociclib arm versus 14.4 months in the capecitabine arm (HR 0.659; 95% CI, 0.437–0.994; p=0.0235) (76).

However, findings from the phase III PEARL trial, which compared palbociclib plus fulvestrant or exemestane to capecitabine in AI-resistant HR+ mBC, showed no efficacy advantage in patients with ESR1 mutations, despite the more favorable toxicity profile of the endocrine-based regimens. These results underscore the importance of individualized treatment sequencing, taking into account both the agent’s mechanism of action and the tumor’s molecular profile (77).

This issue has increasingly been recognized as crucial for managing therapy resistance and optimizing patient outcomes. The PADA-1 trial provided compelling evidence supporting a molecularly guided approach by demonstrating that early intervention based on the detection of rising ESR1 mutations in circulating tumor DNA (bESR1mut) significantly prolonged PFS. Patients who switched from AI plus palbociclib to fulvestrant plus palbociclib upon bESR1mut detection—before radiographic progression—achieved a median PFS of 11.9 months compared to 5.7 months in those who continued AI and palbociclib (HR 0.61; p=0.004). Importantly, this trial underscored the feasibility and clinical benefit of guiding treatment decisions using molecular dynamics rather than waiting for standard radiologic progression, offering a paradigm shift in the timing and duration of therapy in HR+/HER2– mBC (78).

In contrast, the TRINITI-1 and PACE studies explored rational combination strategies for patients progressing after CDK4/6i therapy, with a focus on overcoming endocrine resistance through additional pathway targeting or immune modulation.

TRINITI-1 evaluated a triplet regimen of ribociclib, everolimus, and exemestane in heavily pretreated HR+/HER2– mBC patients. Notably, those with wild-type ESR1 and PIK3CA tumors derived greater benefit (median PFS 9.9 *vs*. 3.2 months), underscoring the role of molecular context in response to targeted combinations (79).

The PACE trial tested the addition of avelumab to fulvestrant with or without palbociclib in the post-CDK4/6i setting. Although PFS was not significantly improved, the triplet arm (avelumab + fulvestrant + palbociclib) showed the most favorable OS outcomes (42.5 months), suggesting potential benefit of immunotherapy-enhanced endocrine strategies in a subset of resistant patients (80).

Together, these studies highlight two complementary but distinct approaches in managing CDK4/6i resistance: one centered on molecular surveillance and dynamic treatment switching (e.g., PADA-1), and the other exploring post-progression therapeutic intensification through rational combinations (e.g., TRINITI-1, PACE). While these strategies focus on optimizing outcomes once resistance has developed, another critical question is whether all patients benefit equally from early CDK4/6i use in the first-line setting.

Addressing this, the phase III SONIA trial examined the sequencing of CDK4/6 inhibition in endocrine-sensitive HR+/HER2– mBC. Specifically, it evaluated whether initiating treatment with a CDK4/6 inhibitor plus an AI provided superior benefit compared to deferring CDK4/6i until after progression on AI monotherapy. The results showed no statistically significant time to second progression (PFS2) advantage with upfront CDK4/6i use (31.0 *vs*. 26.8 months; HR 0.87; p=0.10), despite increased toxicity and higher treatment burden (81). These findings question the universal application of first-line CDK4/6i and highlight the importance of tailoring treatment initiation to individual patient context.

Building on this, several studies have evaluated the effectiveness of continuing or reintroducing CDK4/6 inhibitors after progression. The phase III postMONARCH trial demonstrated that abemaciclib plus fulvestrant reduced the risk of disease progression by 27% (HR 0.66; p=0.01), with benefits observed across subgroups, including patients with ESR1 or PIK3CA mutations (57, 82). Likewise, the phase II MAINTAIN trial showed that switching the endocrine partner while continuing CDK4/6 inhibition with ribociclib significantly improved PFS, with a median of 5.29 months versus 2.76 months for placebo (HR 0.57; p=0.006). Improved 6- and 12-month PFS rates further supported this strategy (83).

Given the variability in trial outcomes, the clinical value of continuing CDK4/6 inhibition beyond progression remains a subject of ongoing debate. Emerging evidence suggests that the distinct pharmacologic and biological profiles of individual CDK4/6 inhibitors may shape patterns of resistance and efficacy. This is exemplified by the divergent results seen in the adjuvant setting: ribociclib demonstrated a significant benefit in the phase III NATALEE trial (83), while palbociclib failed to improve outcomes in both the PALLAS (84) and PENELOPE-B (85) trials. Additionally, abemaciclib has distinguished itself with single-agent activity and favorable results in the nextMONARCH trial when combined with tamoxifen, reinforcing its potential utility in later lines (86). These differences underscore the need for a more nuanced, agent-specific approach to sequencing and rechallenge strategies, ideally guided by molecular profiling and individual tumor biology.

Ongoing trials, including ELAINE 3 and CAPItello-292, as well as the recently published INAVO-120, aim to provide further insights into optimal sequencing and resistance management for CDK4/6-based therapy, but will be discussed in a separate section.

### mTOR inhibitors

The PI3K/AKT/mTOR pathway plays a critical role in both genomic and non-genomic ER signaling and is frequently upregulated in endocrine-resistant HR+/HER2– mBC. This has led to the investigation of mTOR inhibitors as a means to restore endocrine sensitivity. Early efforts focused on temsirolimus, which, in a subgroup of patients under 65, demonstrated a PFS benefit when combined with letrozole (median PFS 9 *vs*. 5.6 months; HR 0.75; p=0.009) (38).

Subsequently, everolimus—an oral allosteric mTOR inhibitor with a superior pharmacological profile—was evaluated in several pivotal trials. The BOLERO-4 phase II study established everolimus combined with letrozole as an active first-line strategy in ET-naïve HR+/HER2– mBC, reporting a median PFS of 22 months, though with considerable toxicity (stomatitis, diarrhea, weight loss, anemia) (87). In the endocrine-resistant setting, BOLERO-2 confirmed the efficacy of everolimus with exemestane versus placebo (median PFS 7.8 *vs*. 3.2 months; HR 0.45; p<0.0001) however, without having shown an overall survival benefit (24). Similarly, other trials demonstrated clinical benefit with everolimus combined with tamoxifen or fulvestrant, with TAMRAD study reporting a 6-month clinical benefit rate of 61% versus 42% with tamoxifen alone (88) while the MANTA trial reported a PFS of 12.3 months for everolimus combined with fulvestrant and 7.6 months for vistusertib combined with fulvestrant (88).

The MIRACLE phase II trial extended this concept to premenopausal patients, showing that everolimus plus letrozole (with goserelin) significantly improved median PFS (19.4 *vs*. 12.9 months; HR 0.64; p=0.008) and clinical benefit rate (72.7% *vs*. 47.5%) compared to letrozole alone. Interestingly, patients who crossed over to everolimus after progression still gained a median PFS of 5.5 months, emphasizing potential utility even in delayed sequencing (89).

More recently, the role of mTOR inhibition post-CDK4/6i progression has garnered attention. A real-world cohort study involving 161 patients treated with everolimus plus various ETs after CDK4/6i failure (primarily exemestane or fulvestrant) demonstrated a median PFS of 6.0 months, with longer benefit observed in patients with prior CDK4/6i exposure ≥18 months (8.7 months), no visceral disease (8.0 months), or who were chemotherapy-naïve in the metastatic setting (7.2 months) (90). These findings are particularly relevant when contrasted with endocrine monotherapy post-CDK4/6i, where median PFS typically ranges from 2.8 to 4.8 months, suggesting that everolimus-based combinations may offer a clinically meaningful alternative in selected patients.

Despite this promise, not all studies were positive. A phase I/IIa study of exemestane, everolimus, and palbociclib in CDK4/6i-pretreated HR+/HER2– mBC patients failed to meet its primary endpoint of clinical benefit rate, achieving just 18.8%, although median OS reached 24.7 months. Multi-omic profiling in this study revealed resistance mechanisms including ESR1, HER2, and BRAF alterations with high RTK/MAPK pathway activity, hinting at molecular subsets more or less likely to benefit (91).

Together, this body of evidence underscores the importance of biologically guided therapy selection. The activity of everolimus appears preserved even after CDK4/6i exposure, especially in patients without visceral crisis or prior chemotherapy, and its utility may be enhanced in the presence of certain molecular aberrations. Future strategies will likely rely on integrative biomarker-driven approaches to define the optimal placement of mTOR inhibitors in the evolving treatment landscape of endocrine-resistant HR+/HER2– mBC.

### Phosphoinositide 3 kinase inhibitors

Aberrant activation of the PI3K/AKT/mTOR pathway is a hallmark of endocrine resistance in HR+/HER2– advanced breast cancer (BC). PI3K mutations, which lead to constitutive pathway activation, are identified in approximately 28%–46% of cases and are consistently associated with a poorer prognosis (92).

Although mTOR inhibitors like everolimus have demonstrated clinical efficacy, their therapeutic potential is limited by compensatory feedback loops that reactivate upstream signaling. One such mechanism involves the IGF-1R–mediated phosphorylation of insulin receptor substrates IRS1 and IRS2 by activated S6K, which reactivates AKT signaling and promotes metabolic adaptation through increased translocation of glucose transporter 4 (GLUT4) to the plasma membrane (38). This facilitates enhanced glucose uptake and accelerates tumor cell metabolism, potentially contributing to the hyperglycemia, ketosis, and insulin dependency observed in some patients receiving mTOR-targeted therapies (93).

To counteract these feedback-driven escape mechanisms, targeted agents directed at upstream nodes of the pathway have been developed. These include isoform-specific PI3K inhibitors, pan-PI3K inhibitors, dual PI3K/mTOR inhibitors, and AKT inhibitors. The latter are broadly categorized into two mechanistic classes: ATP-competitive inhibitors, such as alpelisib, capivasertib, and ipatasertib, which directly inhibit kinase activity at the ATP-binding site; and allosteric inhibitors, such as MK-2206 and miransertib, which bind the pleckstrin homology domain of AKT, preventing its translocation to the plasma membrane and subsequent activation by upstream kinases (94).

Several pan-PI3K inhibitors have been evaluated in combination with ET to overcome resistance in HR+/HER2– mBC particularly in patients harboring PIK3CA mutations. Buparlisib and pictilisib were the most extensively studied agents in this class, with buparlisib advancing to two pivotal phase III trials—BELLE-2 and BELLE-3 (94).

The BELLE-2 trial enrolled patients with AI-resistant disease and prior exposure to CHT. Buparlisib (100 mg daily) combined with fulvestrant modestly improved median PFS compared to fulvestrant plus placebo (6.9 *vs*. 5.0 months; HR 0.78, 95% CI 0.67–0.89; p=0.00021). Importantly, in the subset of patients with confirmed PIK3CA mutations, the benefit was slightly more pronounced (6.8 *vs*. 4.0 months; HR 0.76, 95% CI 0.60–0.97; p=0.014) (95).

BELLE-3 extended these findings to a more heavily pretreated population—postmenopausal women with prior progression on everolimus. Here, buparlisib plus fulvestrant resulted in a median PFS of 3.9 months compared to 1.8 months with fulvestrant alone (HR 0.67, 95% CI 0.53–0.84; p=0.00030) (96).

Despite the moderate improvements in PFS (ranging from 2 to 3 months), the clinical use of buparlisib is limited by its unfavorable toxicity profile. Reported side effects include elevated liver enzymes, hyperglycemia, hypertension, and fatigue, with more severe adverse events such as pleural effusion, dyspnea, and significant liver toxicity observed in 22% of patients in the buparlisib group, compared to 16% in the placebo group (96). However, despite the statistically significant gains, the clinical translation of buparlisib has been hindered by a challenging toxicity profile. Treatment-related adverse events—including transaminitis, hyperglycemia, hypertension, and fatigue—were frequent, with grade ≥3 events such as pleural effusion and liver toxicity occurring in 22% of patients receiving buparlisib, compared to 16% in the placebo arm (96). These safety concerns, coupled with modest efficacy, ultimately limited its therapeutic development and redirected focus toward isoform-selective PI3K inhibitors with more favorable tolerability.

Given the toxicity and limited therapeutic window of pan-PI3K inhibitors, attention has shifted toward isoform-specific agents, particularly those targeting the PI3Kα isoform, which is most frequently mutated in HR+/HER2– BC. Alpelisib, a selective PI3Kα inhibitor, was evaluated in the pivotal phase III SOLAR-1 trial. Among patients with PIK3CA-mutant tumors, the combination of alpelisib and fulvestrant significantly improved PFS compared to fulvestrant alone (11.0 *vs*. 5.7 months; HR 0.65, 95% CI 0.50–0.85; p<0.001) (92). Although the trial did not meet the prespecified threshold for overall survival (OS) benefit in the overall mutant cohort (39.3 *vs*. 31.4 months; HR 0.86; p=0.15), a clinically meaningful OS gain was observed in the subset of patients with visceral disease, particularly those with lung and/or liver metastases (37.2 *vs*. 22.8 months; HR 0.68; 95% CI 0.46–1.00) (92).

Further support for alpelisib’s biological activity was provided by a phase II study of alpelisib monotherapy, which demonstrated that early reductions in PIK3CA mutations detected in circulating tumor DNA (ctDNA) were strongly associated with improved outcomes. Patients with a molecular response by week 8 experienced longer PFS (HR 0.24, 95% CI 0.083–0.67; p=0.0065), and similar associations were seen in those with detectable ESR1 mutations at baseline (HR 0.22, 95% CI 0.078–0.60; p=0.003) (92, 97).

These findings highlight the potential utility of ctDNA as an early predictive biomarker for response to PI3K inhibition, particularly in endocrine-resistant disease.

Despite its efficacy, alpelisib’s clinical use is constrained by a notable toxicity profile. In the SOLAR-1 study, grade ≥3 hyperglycemia occurred in 36.6% of patients, while rash and diarrhea were reported in 9.9% and 6.7%, respectively (98). These adverse events require early intervention and may limit the use of alpelisib in frail patients or those with pre-existing metabolic comorbidities.

Similarly, in the SANDPIPER trial, the PI3K inhibitor taselisib was evaluated in women with recurrent or progressive BC, including those with metastatic disease following treatment with an AI. Patients were randomized to receive either taselisib or placebo in combination with fulvestrant. The taselisib group demonstrated a PFS improvement of 2 months (7.4 months [95% CI, 7.26–9.07] *vs*. 5.4 months [95% CI, 3.68–7.29]; HR 0.70, 95% CI 0.56–0.89; p=0.0037). However, serious adverse events were noted in the taselisib arm compared to the placebo arm (32.0% *vs*. 8.9%) (99).

Considering the toxicity limitations observed with alpelisib and taselisib, attention has expanded toward AKT inhibition as a complementary strategy to disrupt the PI3K/AKT/mTOR axis. Capivasertib, a selective ATP-competitive pan-AKT inhibitor, has demonstrated notable antitumor activity in HR+/HER2– breast cancer across various clinical settings. Early-phase data from the BEECH trial, which combined capivasertib with paclitaxel in endocrine-resistant mBC, did not yield significant improvements in PFS or tolerability. However, this may have been confounded by CHT-related toxicities and the absence of molecular stratification (100).

More encouraging results emerged from endocrine-based combinations. In the phase II FAKTION trial, capivasertib plus fulvestrant significantly improved median PFS (10.3 *vs*. 4.8 months; HR 0.56; p=0.0023) and OS (29.3 *vs*. 23.4 months; HR 0.66; p=0.0035) compared to fulvestrant alone. The benefit was particularly pronounced in patients with PI3K/AKT/PTEN pathway alterations, where median PFS reached 12.8 months versus 4.6 months (HR 0.44; p=0.0014), and median OS extended to 38.9 months compared to 20.0 months with placebo (HR 0.46; p=0.0047) (22).

While capivasertib was generally well tolerated, treatment-related grade 3/4 toxicities such as hyperglycemia (20–24%), diarrhea (14–17%), and rash (11–16%) were not uncommon, underscoring the importance of adverse event monitoring.

Building on these findings, the phase III CAPItello-291 trial confirmed the benefit of AKT inhibition in a more heterogeneous, heavily pretreated population. In this study, capivasertib plus fulvestrant doubled PFS compared to placebo (7.2 *vs*. 3.6 months; HR 0.60; p<0.001), with even greater efficacy in patients harboring AKT-pathway aberrations (7.3 *vs*. 3.1 months; HR 0.50; p<0.001) (101).

These results firmly establish capivasertib as a clinically meaningful option, especially in the post-CDK4/6i setting. The ongoing CAPItello-292 trial is now evaluating capivasertib in triplet therapy with palbociclib and fulvestrant, aiming to clarify its role earlier in the treatment sequence.

Most recently, the selective PI3K inhibitor inavolisib has garnered significant attention following the positive findings of the phase III INAVO120 trial. In patients with PIK3CA-mutant, HR+/HER2– advanced breast cancer, the addition of inavolisib to palbociclib and fulvestrant substantially extended PFS to 17.2 months compared to 7.3 months with placebo. Objective response rates were also markedly improved (62.7% *vs*. 28.0%), and according to the most recent ASCO announcement there was also a significant improvement in the OS with 34 months in the experimental, inavolisib arm *vs*. 27 months in the placebo arm (102). Importantly, the safety profile was favorable relative to alpelisib, with lower rates of grade ≥3 hyperglycemia (5.6%) and minimal occurrences of stomatitis or diarrhea. Neutropenia remained the most common high-grade toxicity (80.2%), consistent with palbociclib-related myelosuppression (21). Treatment discontinuation due to adverse events was infrequent (6.8%), suggesting that inavolisib may represent a more tolerable and efficacious next-generation alternative to alpelisib for patients with endocrine-resistant, PIK3CA-mutated disease (21, 94).

Taken together, these data underscore the growing importance of biomarker-guided inhibition of the PI3K/AKT pathway in HR+/HER2– mBC. With multiple agents now demonstrating efficacy in genomically enriched populations, future therapeutic strategies will likely hinge on refined molecular profiling to optimize the timing, combination, and sequencing of targeted therapies.

### Antibody drug conjugates

In HR+ mBC that has become resistant to endocrine and targeted therapies, single-agent CHT remains a standard treatment option. However, its efficacy is modest, with median PFS typically ranging from 6 to 7 months.

Antibody-drug conjugates (ADCs) have emerged as a transformative therapeutic strategy, consisting of monoclonal antibodies directed against tumor-associated antigens, conjugated to cytotoxic payloads. This design allows for the selective delivery of chemotherapy agents to tumor cells expressing the target antigen (103). In BC, the first ADC to gain regulatory approval was ado-trastuzumab emtansine (T-DM1) in 2013 for HER2+ disease, followed by fam-trastuzumab deruxtecan (T-DXd, DS-8201) in 2022 (104).

The therapeutic efficacy of ADCs is influenced by several factors, beyond the payload itself, including the drug to antibody ratio, the level of antigen expression on tumor cells, and quite critically, the nature of the linker (105). Linkers can be either cleavable or non-cleavable, determining the mechanism of intracellular drug release. Non-cleavable linkers require internalization and lysosomal degradation of the ADC for payload release within the targeted cell. In contrast, cleavable linkers, such as those used in T-DXd, enable payload release in response to specific intracellular conditions, such as acidic pH or enzymatic activity (105). Notably, cleavable payloads can diffuse beyond the initially targeted cell, exerting cytotoxic effects on neighboring tumor cells with lower or heterogeneous antigen expression — a phenomenon known as the bystander effect (106). This has been a central hypothesis underlying the evaluation of ADCs in tumors with low or heterogeneous target expression.

In light of these insights, HER2 classification was redefined in 2021 to encompass a broader continuum of HER2 expressions, including HER2-low and ultra-low categories. HER2-0 is now defined by a complete absence of staining in infiltrating tumor cells, HER2 ultra-low by ≤10% of cells with faint or weak membrane staining and HER2-low by an IHC score of 1+ or 2+ with a negative *in situ* hybridization (ISH) result.

This reclassification has significantly expanded the therapeutic landscape for BC, with HER-2 low tumors comprising almost 70% of cases, previously considered HER2-negative tumors, now showing clinical benefit from agents such as T-DXd.

Subsequent genomic studies have supported the clinical distinction of HER2-low tumors. In a large sequencing study of 1,039 HER2- mBC, 47% were reclassified as HER2-low and 53% as HER2-0. While HER2-low tumors exhibited a slightly higher ERBB2 copy count compared to HER2-0 (2.05 *vs*. 1.79), no significant differences were observed in mutational burden or broader genomic alterations, suggesting that HER2-low may not represent a distinct molecular subtype (107). Longitudinal studies of matched primary and recurrent tumor samples further highlight the dynamic nature of HER-2 expression. HER2-low status was identified in 34.2% of primary tumors, rising to 37.3% in recurrent lesions. Notably, about 38% of patients exhibited shifts in HER2 expression over time, most frequently transitioning between HER2–0 and HER2-low (108).

What truly supported this paradigm shift were its clinical implications, as evidenced by the DESTINY-Breast 04 and DESTINY-Breast 06 trials, which demonstrated that patients with low and ultra-low HER2 expression can benefit from HER2-directed therapy.

In the DESTINY-Breast04, a pivotal phase III trial, 557 patients with HER2-low mBC were enrolled, of whom 494 (88.7%) were HR+ tumors and 63 (11.3%) HR-. Among patients with HR+ tumors, T-DXd significantly improved median PFS to 10.1 months compared to 5.4 months with CHT (HR 0.51; p<0.001) and extended OS to 23.9 months compared to 17.5 months (HR 0.64; p=0.003). Across the entire cohort, median PFS and OS were also superior with T-DXd: 9.9 months versus 5.1 months (HR 0.50; p<0.001), and 23.4 months versus 16.8 months (HR 0.64; p=0.001), respectively. The ORR in HR+ patients reached 52.6% with T-DXd compared to 16.3% with CHT. Grade 3 or higher adverse events occurred in 52.6% of T-DXd-treated patients versus 67.4% with CHT, with T-DXd being associated with interstitial lung disease (ILD) or pneumonitis in 12.1% of patients (109). Similarly, results were favorable in patients without previous CHT for metastatic disease, in both the HER2-low and ultralow categories, as illustrated through the DESTINY-Breast 06 phase 3 trial. In the HER2-low group, T-DXd significantly extended median PFS to 13.2 months compared to 8.1 months with CHT (HR 0.62; p<0.001), while in the ultralow category median PFS was similar 13.2 months *vs*. 8.3 months in the physician’s choice of chemotherapy (HR 0.72). However, OS data is still immature. Grade 3 or higher adverse events occurred in 52.8% of T-DXd patients versus 44.4% in the CHT group, with ILD or pneumonitis reported in 11.3% of T-DXd patients (110).

The DAISY phase 2 trial further provided mechanistic insights into the differential efficacy of T-DXd across HER2 expression levels. ORR was highest in HER2-overexpressing tumors (70.6%) with a median PFS of 11.1 months, while HER2-low and HER2–0 tumors demonstrated ORRs of 37.5% and 29.7%, and PFS of 6.7 and 4.2 months, respectively. Tumor shrinkage in target lesions reflected this gradient, with median reductions of 57% in HER2-positive, 25% in HER2-low, and 12.5% in HER2-0 cohorts, respectively (111).

Beyond DESTINY-Breast04 and DESTINY-Breast06, additional trials have reinforced the versatility of T-DXd across distinct subpopulations in advanced BC. The DESTINY-Breast07 trial evaluated T-DXd alone or in combination with pertuzumab in the first-line setting for HER2-positive mBC. Both treatment arms demonstrated high objective response rates (77–82%) and sustained clinical benefit, with 12-month PFS rates nearing 90% in the combination arm (112). These findings support the feasibility of T-DXd-based doublet regimens and inform the design of ongoing first-line studies such as DESTINY-Breast09.

The DESTINY-Breast12 trial addressed a critical unmet need by prospectively assessing the efficacy of T-DXd in patients with HER2-positive mBC and brain metastases—a population frequently underrepresented in clinical trials. T-DXd showed meaningful intracranial activity, with a 12-month PFS of 61.6% and central nervous system (CNS)-specific PFS of 58.9% in patients with brain metastases. In patients without CNS involvement, the systemic ORR reached 62.7% (113). All these findings support the use of T-DXd regardless of the presence or activity of brain metastases.

Collectively, these trials underscore the expanding therapeutic footprint of T-DXd—from HER2-low and ultralow hormone receptor-positive disease to HER2-positive tumors with CNS involvement—highlighting its potential as a foundational agent across molecular and clinical subtypes in advanced BC.

Beyond HER2-directed therapy, ADCs have shown promise in other molecular contexts. In heavily pretreated patients, sacituzumab govitecan (SG), an ADC targeting trophoblast cell-surface antigen-2 (Trop-2) and linked via a hydrolysable linker to SN-38, demonstrated robust activity in pretreated HR+, HER2− mBC patients. Internalization is not required, as hydrolysis can occur in the tumor microenvironment, contributing to the bystander effect (104).

The TROPiCS-02 trial showed a significant OS benefit with SG compared to CHT (median 14.4 *vs*. 11.2 months; HR 0.79; p=0.020), with consistent efficacy across different levels of Trop-2 expression. The ORR was also superior with SG (21% *vs*. 14%; OR 1.63; p=0.035), and the safety profile remained manageable (114).

Datopotamab deruxtecan (Dato-DXd), a novel Trop-2 directed ADC using the same cleavable linker and topoisomerase I inhibitor payload as T-DXd, is currently under investigation. In the TROPION-Breast01 trial, Dato-DXd significantly improved PFS (6.9 *vs*. 4.9 months; HR 0.63, p<0.0001) and demonstrated a higher ORR (36.4% *vs*. 22.9%) compared to CHT in patients who experienced disease progression on ET and had undergone up to two prior lines of CHT in the metastatic setting. Notably, Dato-DXd was associated with fewer grade ≥3 treatment-related adverse events (20.8% *vs*. 44.7%), suggesting an improved safety profile over conventional cytotoxic regimens (115).

### Poly ADP-ribose polymerase inhibitors

In BRCA-mutated BC, loss of homologous recombination repair renders tumor cells unable to effectively repair double-strand DNA breaks. PARP inhibitors (PARPi) capitalize on this vulnerability by blocking the repair of single-strand breaks, through inhibition of PARP enzymes. During DNA replication, these unresolved single-strand breaks are converted into double-strand breaks, which accumulate and lead to cell death in the context of BRCA deficiency. This forms the mechanistic basis for PARPi as a targeted therapy for BC with DNA repair deficiencies (115).

In HR+ mBC patients with germline BRCA1 or BRCA2 mutations, PARPi such as olaparib and talazoparib, have demonstrated clinical efficacy in pivotal trials. The OlympiAD trial reshowed that olaparib significantly improved PFS over CHT (7.0 months *vs*. 4.2 months) in patients with HER2- germline BRCA-mutated mBC, including HR+ subtypes. In first-line settings, olaparib extended median OS to 22.6 months versus 14.7 months with CHT (HR = 0.55; 95% CI, 0.33–0.95), with 3 year survival rates of 40.8% compared to 12.8% (116). Similarly, the phase 3 EMBRACA trial demonstrated that talazoparib significantly prolonged median PFS of 8.6 months compared to 5.6 months with CHT (HR = 0.54; 95% CI, 0.41–0.71; p < 0.0001) and achieved a higher ORR of 62.6% versus 27.2%. While talazoparib was associated with increased hematologic toxicity, particularly grade 3–4 anemia (55% *vs*. 38%), it was linked to improved quality of life and delayed symptom deterioration compared to CHT (117).

Following these findings, PARPi are currently recommended as second-line therapy after progression on CDK4/6i and ET in patients with BRCA germline mutations. Given the significant OS advantage associated with CDK4/6i, these agents are generally prioritized before PARP inhibition, as no OS benefit was observed with PARPi alone (40). Nevertheless, the optimal sequencing of therapies continues to be a topic of active debate among clinicians and researchers.

### PROteolysis TArgeting Chimeras

PROteolysis Targeting Chimeras (PROTACs), alongside lysosome targeting chimeras (LYTACs), represent an innovative class of targeted protein degradation therapies. These heterobifunctional molecules consist of two ligands: one that binds the protein of interest, and one that recruits E3 ubiquitin ligase, connected by a flexible linker. The formation of this ternary complex promotes polyubiquitination of the target protein, leading to its proteasomal degradation. Unlike traditional inhibitors, PROTACs eliminate rather than inhibit their targets, offering a strategy to overcome resistance driven by receptor overexpression or mutation.

Since their conceptualization in 2001, PROTACs targeting ER for BC have advanced considerably. ARV-471 (vepdegestrant), a novel oral ER-targeting PROTAC, recently received FDA fast-track designation for monotherapy in endocrine-resistant mBC (118), based on results from the NCT04072952 and the VERITAC clinical trials.

In the phase 1b NCT04072952 trial, vepdegestrant combined with palbociclib demonstrated a clinical benefit rate of 63.0% (95% CI, 47.5%-76.8%) for HR+ mBC. Subgroup analyses revealed even higher activity in ESR1-mutant tumors (CBR 72.4%) versus ESR1 wild-type (CBR 53.3%). Median PFS was 11.2 months (95% CI, 8.2-16.5) overall, extending to 13.7 months (95% CI, 8.2–not reached) in ESR1-mutant patients and 11.1 months (95% CI, 2.8-19.3) in ESR1 wild-type patients (119).

In the monotherapy VERITAC study which enrolled heavily pretreated ER+/HER2− mBC patients, ARV-471–200 mg QD achieved a CBR of 37.1% (95% CI: 21–55) overall and 47.4% (95% CI: 24–71) in those with mutant ESR1. Median PFS was 3.5 months (95% CI: 1.8–7.8). The safety profile was favorable, with most adverse events being grade 1–2 and including fatigue (40%), hot flushes (17%), and nausea (14%), supporting its advancement to phase III evaluation (120).

Just presented at ASCO 2025, the results of the pivotal phase III randomized, open-label trial comparing vepdegestrant (200 mg) with fulvestrant further underscore its clinical potential. In this study, 624 patients with ER+/HER2− advanced BC previously treated with a CDK4/6 inhibitor and up to two lines of ET were randomized. Among the 270 patients harboring ESR1 mutations, vepdegestrant significantly improved median PFS to 5.0 months (95% CI, 3.7–7.4) versus 2.1 months (95% CI, 1.9–3.5) with fulvestrant (HR 0.58; 95% CI, 0.43–0.78; P<0.001). In the overall population, median PFS was 3.8 months *vs*. 3.6 months (HR 0.83; 95% CI, 0.69–1.01; P=0.07). Grade ≥3 adverse events were reported in 23.4% of patients in the vepdegestrant group and in 17.6% of those receiving fulvestrant, with low rates of treatment discontinuation (2.9% *vs*. 0.7%) (121).

**Figure 6:**
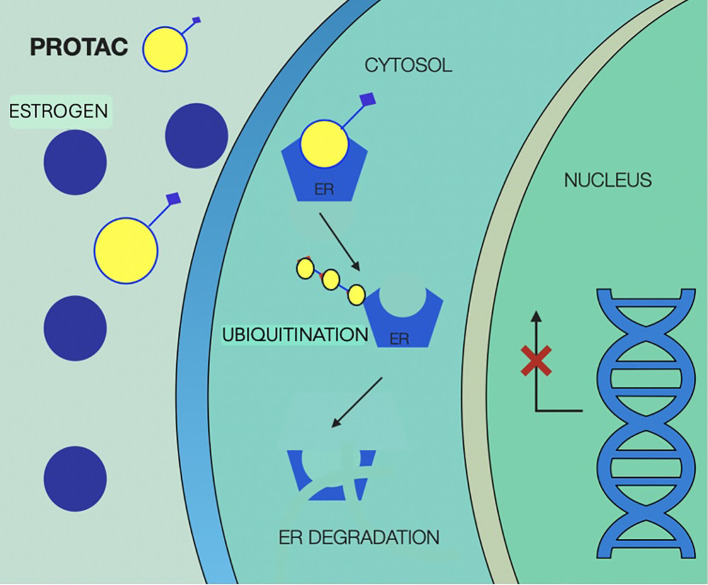
Mechanism of action of Proteolysis Targeting Chimera agents (PROTACs), illustrating selective degradation of the estrogen receptor (ER). PROTAC molecules simultaneously bind ER and recruit an E3 ubiquitin ligase, forming a ternary complex that leads to polyubiquitination of the receptor. This post-translational modification marks ER for proteasomal degradation, resulting in the complete elimination of both ligand-bound and mutant receptor species. PROTAC, proteolysis targeting chimera; ER, estrogen receptor.

Mechanism of action of PROTACs are illustrated in **Figure 6**.

### Complete estrogen receptor antagonists

The complete estrogen receptor antagonists (CERANs) are a distinct class of small molecules designed to comprehensively inhibit both activation domains, AF1 and AF2, of the ER. Their mechanism involves degradation of ER, functional silencing through co-repressors recruitment, such as nuclear receptor co-repressor (N-CoR) binding to AF1, and consequently inhibition of ER-driven transcription and tumor proliferation (118).

Among agents in this category, OP-1250 has emerged as an orally bioavailable CERAN with selective ER degradation (SERD) activity. In preclinical studies, OP-1250 effectively inhibited and degraded both wild-type and mutant ER. Early clinical evaluation in a phase I/II dose-escalation and expansion trial showed promising results. At the recommended phase II dose, OP-1250 achieved in heavily pretreated HR+ mBC patients, an ORR of 18% and a CBR of 38% (26, 118).

**Figure 7:**
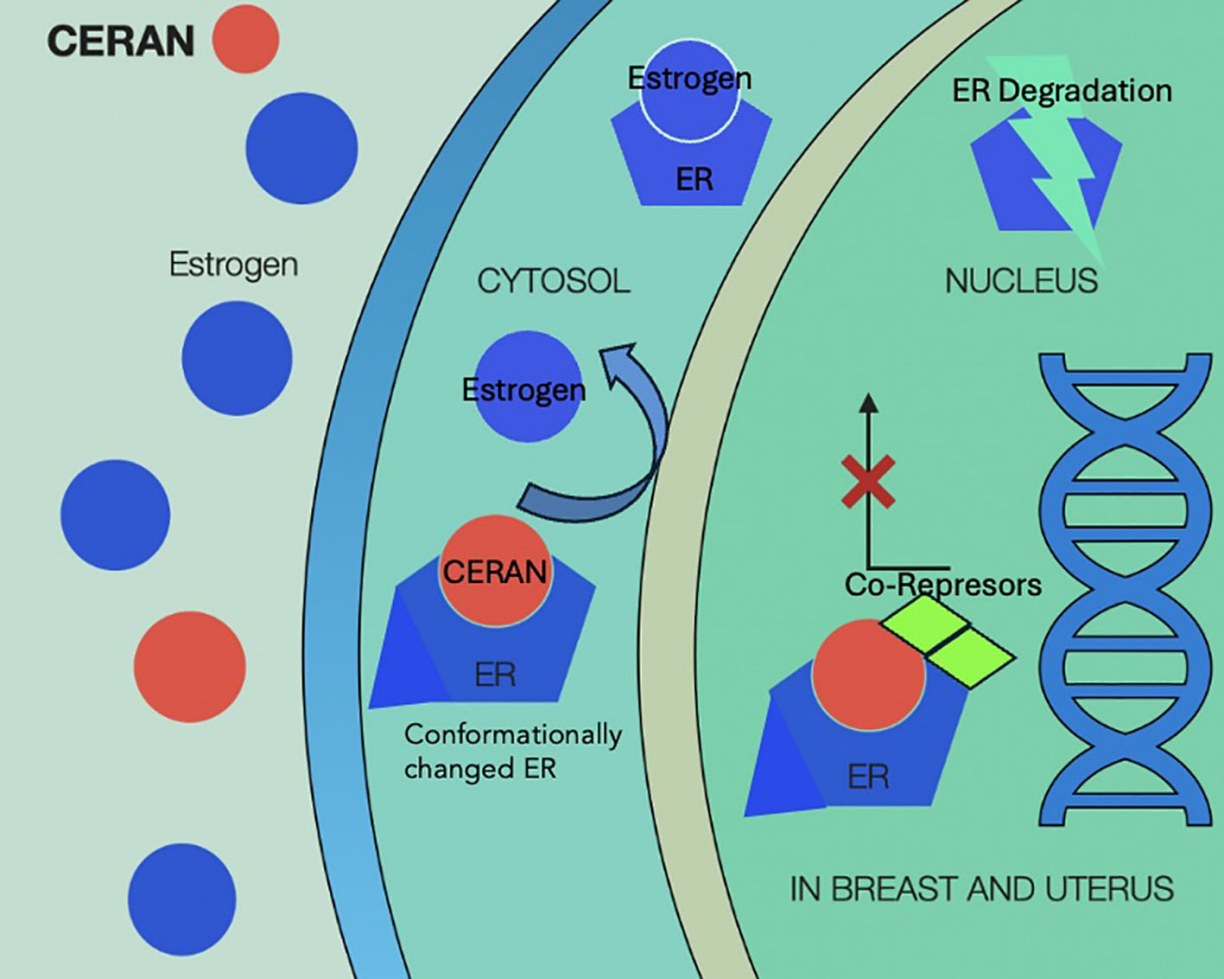
Mechanism of action of complete estrogen receptor antagonists (CERANs) showing competitive binding to the estrogen receptor (ER) in the cytosol, displacement of endogenous estrogen, and inhibition of ER activation. Upon CERAN binding, the receptor fails to undergo appropriate conformational change, leading to co-repressor recruitment, nuclear translocation blockade, or transcriptional silencing. This results in ER degradation and suppression of estrogen-driven gene expression in target tissues. CERAN, complete estrogen receptor antagonist; ER, estrogen receptor.

CERANs mechanism of action is illustrated in **Figure 7**.

### Selective estrogen receptor covalent antagonists

Selective Estrogen Receptor Covalent Antagonists (SERCAs) are an innovative class of ER targeting agents that inactivate ER by covalently binding to cysteine 530 (C530), a non-conserved residue within the ligand-binding pocket of ER alpha. This mechanism effectively antagonizes both wild-type and mutant ER receptors, including clinically relevant ESR1 mutations associated with endocrine resistance (26).

H3B-5942 was the first-in-class experimental oral compound with high selectivity for C530 demonstrating superior antitumor activity to fulvestrant in preclinical BC xenograft models with both ESR1 Y37S mutation and ESR1 wild type. Research around this agent paved the way for development of H3B-6545, a next generation SERCA currently under clinical investigation in multiple trials for ET-resistant HR+ BC (clinical trials: NCT03250676, NCT04568902, NCT04288089) (118). In a phase I/II trial H3B-6545 demonstrated an objective response rate (ORR) of 16.4% and a clinical benefit rate (CBR) of 39.7% in a heavily pretreated population (n=94), most of whom had prior CDK4/6 inhibitor exposure. Median PFS was 3.8 months, with responses enriched in patients with visceral disease and ESR1 mutations. Adverse events included bradycardia (asymptomatic in 35%, grade ≥2 in 5%), gastrointestinal symptoms, cytopenias, and renal function changes. Serious adverse events occurred in 21% of patients, leading to treatment discontinuation in 13% (26).

H3B-6545 is also being evaluated in combination with palbociclib in patients with HR+ mBC after ≥2 prior treatment lines (NCT04288089), further supporting its potential as a next-generation endocrine backbone in resistant disease.

**Figure 8:**
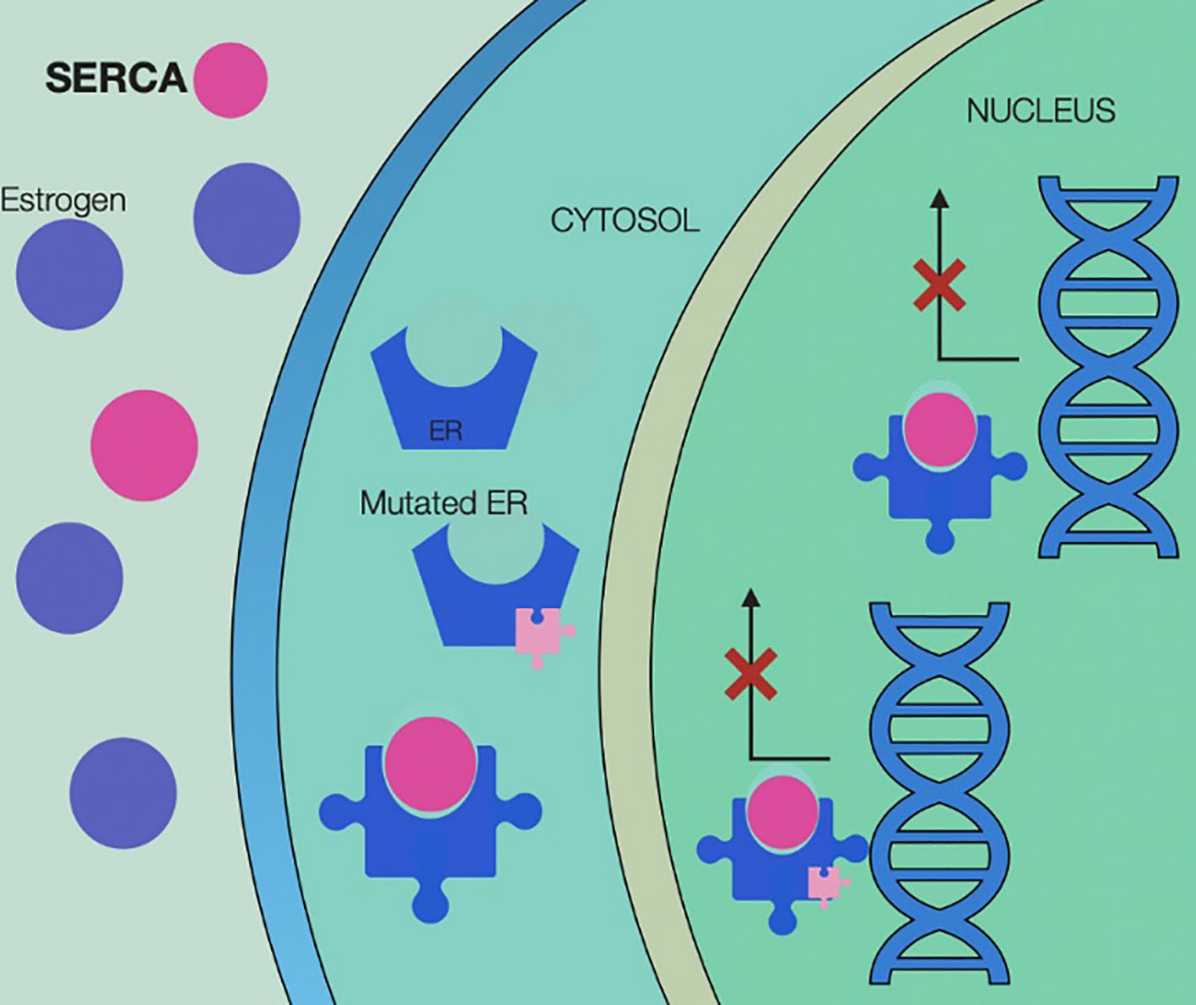
Selective estrogen receptor covalent antagonists (SERCA) mechanism of action, illustrating irreversible inhibition of both wild-type and mutant estrogen receptors (ER) through covalent binding at cysteine 530 within the ligand-binding domain. By forming a covalent bond, SERCAs prevent receptor activation, block estrogen binding, and disrupt transcriptional activity—even in the presence of activating ESR1 mutations. This results in sustained receptor inactivation, suppression of estrogen-driven gene expression, and inhibition of tumor cell proliferation. *SERCA, Selective Estrogen Receptor Covalent Antagonists*; *ER, estrogen receptor*.

Mechanism of action of SERCAs are illustrated in **Figure 8**.

### Selective Human Estrogen Receptor Partial Agonist

Selective Human Estrogen Receptor Partial Agonists (ShERPAs) represent a novel class of benzothiophene-derived compounds engineered to selectively modulate ER signaling with partial agonist activity. These agents exbibit nanomolar potency in BC cell lines and are designed to mimic estradiol activity by binding ER in the nucleus, promoting its translocation to extranuclear compartments, and ultimately disrupting proliferative signaling. In preclinical models, several ShERPAs have shown significant antitumor activity, including in xenografts of endocrine-independent tamoxifen-resistant BC (122). Specifically, the compounds BMI-135 and TTC-352 have emerged as leading drugs within their category. Notably, TTC-352 has progressed to a phase I clinical trial for HR+ BC resistant to ET, demonstrating a favorable safety profile alongside early signs of antitumor efficacy (118).

### Future directions

Although development of immune checkpoint inhibition strategies (ICI) targeting tumor-infiltrating lymphocytes (TILs) within the tumor microenvironment has revolutionized cancer treatment, patients with HR+/HER2- BC have derived limited benefit. This is primarily due to the unique pathophysiological characteristics of the tumor microenvironment in HR+/HER2- BC, which is characterized by low levels of TILs, reduced expression of HLA class I molecules (123), and the recruitment of immunosuppressive cells such as macrophages and regulatory T cells (Tregs). These factors render HR+/HER2- BC less responsive to conventional immunotherapies and are associated with a poorer prognosis (124).

However, ICI targeting the programmed death receptor 1 (PD-1) pathway, along with other immunotherapeutic approaches such as CTLA-4 monoclonal antibodies (e.g., ipilimumab), have been explored in HR+ BC, with several clinical trials still underway.

In the KEYNOTE-028 study, pembrolizumab achieved an ORR of 12% in heavily pretreated PD-L1-positive HR+/HER2− BC patients, albeit with prolonged response durations in some cases. These findings highlight the challenges of achieving significant clinical benefit with ICIs, especially in heavily pretreated HR+/HER2- BC populations (125). Similarly, avelumab demonstrated an ORR of only 3% in a broader mBC population, including 43% with HR+/HER2− disease, again underscoring the limitations of ICIs in this group (126).

Preclinical data suggest that CDK4/6 inhibitors may sensitize tumors to ICIs by reducing Treg populations, downregulating PD-1 expression, enhancing antigen presentation, and promoting cytotoxic T cell activity. This has spurred clinical investigations into ICI–CDK4/6i combinations, sometimes paired with ET (20). A phase 1b trial evaluating abemaciclib plus pembrolizumab—with or without anastrozole—was terminated early due to hepatotoxicity and pneumonitis, despite preliminary antitumor activity (127). Conversely, a small open-label trial with palbociclib, pembrolizumab, and letrozole demonstrated encouraging responses with 31% CR and 25% PR among patients who received pembrolizumab upfront. When pembrolizumab was added later in the treatment sequence, the CR and PR rates increased to 50% (20, 128).

These findings underscore the need for further research to validate the potential benefits of ICIs combination therapy in HR+/HER2- BC.

Beyond immunotherapy, proteasome inhibitors such as bortezomib are being reevaluated in HR+/HER2− BC for their ability to destabilize ER signaling and enhance the efficacy of ET (129).

Antiprogestins—including selective progesterone receptor antagonists like onapristone—have also emerged as potential options in endocrine-resistant disease. Targeting additional signaling pathways beyond ER remains an important strategy to overcome endocrine resistance. Notably, ER activation can occur independently of ligand binding through the Notch signaling axis, leading to ERα dysregulation and sustained transcriptional activity even in the absence of estrogen. Inhibiting the Notch pathway has therefore emerged as a promising approach to restore ET sensitivity. Similarly, overactivation of AURKA contributes to ERα destabilization and downregulation, further driving resistance. The AURKA inhibitor alisertib is currently under investigation in this context. Another emerging strategy involves modulation of the JAK-STAT pathway via the lymphocyte adaptor protein LNK, which plays a role in hormone receptor signaling and immune cross-talk. Together, these pathways—Notch, AURKA, and JAK/STAT—represent rational and biologically distinct targets under exploration for restoring hormone sensitivity in resistant HR+ breast cancers (130).

Artificial intelligence (AI) is poised to accelerate progress across several domains in HR+/HER2− BC. In drug discovery, AI-based platforms have been utilized to predict the binding affinity of SERDs and to identify synergistic combinations with CDK4/6i by integrating pharmacogenomic and transcriptomic datasets. In prognostication, AI-enhanced digital pathology tools—such as those developed by PathAI and Paige—have demonstrated the ability to predict recurrence risk and treatment response in ER+ BC by analyzing H&E-stained histology slides in conjunction with molecular profiles (131). For instance, a study introduced the BRACE marker, derived from AI-based assessment of tumor heterogeneity in H&E images, which effectively stratified early-stage luminal/HER2-negative BC patients for distant metastasis-free survival and BC-specific survival, showing comparable prediction accuracy to traditional prognostic indices (132). Additionally, circulating tumor DNA (ctDNA) analysis, interpreted through AI algorithms, has enabled earlier detection of ESR1 mutations and prediction of endocrine resistance. Notably, the PADA-1 trial utilized ctDNA monitoring to detect ESR1 mutations, allowing for timely therapeutic interventions (133). In therapeutic delivery, AI algorithms are now being piloted to optimize CDK4/6 inhibitor dosing based on patient-specific hematologic trends and toxicity profiles, reducing the incidence of treatment interruptions and improving adherence. Collectively, these advances support the integration of AI as a transformative tool for precision oncology in HR+/HER2− mBC, with the potential to refine prognostic models, enhance therapeutic targeting, and personalize treatment delivery.

## Discussion

The therapeutic landscape for HR+/HER2- mBC has evolved markedly with the integration of targeted therapies and next-generation endocrine strategies. While CDK4/6i combined with ET remain foundational in first-line treatment, resistance – particularly via ESR1 mutations – continues to drive disease progression and poses a major clinical challenge.

The EMERALD trial established the efficacy of oral selective estrogen receptor degraders in this space. Elacestrant significantly improved PFS over standard endocrine therapy in patients with ESR1 mutations, leading to its regulatory approval. Building on this, the EMBER-3 phase III trial demonstrated that imlunestrant, another oral SERD, achieved a statistically significant progression-free survival benefit in both ESR1-mutant and wild-type populations following prior CDK4/6 inhibitor exposure, while also offering a favorable safety profile.

Most recently, findings from the SERENA-6 trial showed compelling data on camizestrant. This biomarker-driven, ctDNA-guided study showed that early intervention with camizestrant—upon detection of rising ESR1 mutations prior to radiologic progression—delayed disease progression compared to standard AI continuation. This represents the first phase III evidence supporting a molecular response–adapted strategy in HR+ HER2- mBC and positions camizestrant as a viable option not only for resistant disease but potentially in mutation-interceptive protocols.

In parallel, the INAVO120 trial has reaffirmed the clinical value of targeting the PI3K/AKT pathway. In patients with PIK3CA-mutant tumors, the addition of inavolisib to palbociclib and fulvestrant nearly doubled median PFS versus placebo, with manageable toxicity. These findings validate a biomarker-driven approach post-CDK4/6i failure and support the sequencing of targeted pathway inhibitors in genomically defined subgroups.

The therapeutic horizon is also expanding with ADCs. Initially positioned in later-line settings, agents like T-DXd and sacituzumab govitecan are now being evaluated—and in some cases approved—for earlier lines of treatment, including post-CDK4/6i and even in the first line setting for select high-risk populations. Their impressive activity in HER2-low and Trop-2–positive disease may soon redefine standard treatment sequences.

Efforts to enhance immunotherapy efficacy are also ongoing, though the immunologically “cold” tumor microenvironment typical of HR+, HER2-negative tumors remains a barrier. Trials combining checkpoint inhibitors with CDK4/6i or ET have yielded mixed results, but certain histologies, such as lobular carcinoma, may harbor enhanced sensitivity.

Finally, AI is emerging as a disruptive tool across drug discovery, prognostic modeling, and treatment personalization. AI-enabled pathology markers such as BRACE, ctDNA analytics for ESR1 dynamics, and predictive dosing algorithms for CDK4/6i are early examples of how machine learning is transforming care in this setting. These technologies offer unprecedented granularity in treatment adaptation, accelerating the shift toward dynamic, biomarker-informed precision oncology.

## Conclusion

The management of HR+ HER2− mBC is undergoing a paradigm shift, driven by biomarker-informed treatment selection, adaptive therapy strategies, and integration of emerging technologies. Oral SERDs such as elacestrant, imlunestrant, and camizestrant are reshaping endocrine resistance management, with SERENA-6 demonstrating the feasibility of ctDNA-guided early intervention. The INAVO120 trial highlights the value of targeting the PI3K/AKT pathway in genomically defined subgroups, while ADCs are gaining traction beyond second line use and are being positioned for earlier implementation.

As combination approaches evolve, incorporating ET, specific pathway inhibitors, ADCs, and immunotherapies into rational regimens will be key. AI will play an increasingly central role in optimizing these strategies—through drug discovery, real-time monitoring, and personalized dosing—to achieve maximum clinical benefit.

Ultimately, future advances in HR+ mBC will rely on the convergence of molecular diagnostics, translational research, and AI-enabled tools to deliver precision oncology solutions that will not only overcome resistance but will be proactively designed to prevent it.
